# Evidence that protein thiols are not primary targets of intracellular reactive oxygen species in growing *Escherichia coli*

**DOI:** 10.3389/fmicb.2023.1305973

**Published:** 2023-12-13

**Authors:** Stefanie S. Eben, James A. Imlay

**Affiliations:** Department of Microbiology, University of Illinois, Urbana, IL, United States

**Keywords:** oxidative stress, glutaredoxin, thioredoxin, *E. coli*, disulfide bond formation

## Abstract

The oxidizability of cysteine residues is exploited in redox chemistry and as a source of stabilizing disulfide bonds, but it also raises the possibility that these side chains will be oxidized when they should not be. It has often been suggested that intracellular oxidative stress from hydrogen peroxide or superoxide may result in the oxidation of the cysteine residues of cytoplasmic proteins. That view seemed to be supported by the discovery that one cellular response to hydrogen peroxide is the induction of glutaredoxin 1 and thioredoxin 2. In this study we used model compounds as well as alkaline phosphatase to test this idea. Our results indicate that molecular oxygen, superoxide, and hydrogen peroxide are very poor oxidants of N-acetylcysteine and of the protein thiols of alkaline phosphatase *in vitro*. Copper could accelerate thiol oxidation, but iron did not. When alkaline phosphatase was engineered to remain in the cytoplasm of live cells, unnaturally high concentrations of hydrogen peroxide were required to oxidize it to its active, disulfide-dependent form, and toxic levels of superoxide had no effect. At the same time, far lower concentrations of these oxidants were sufficient to poison key metalloenzymes. The elimination of glutaredoxin 1 and thioredoxin 2 did not change these results, raising the question of why *E. coli* induces them during peroxide stress. In fact, when catalase/peroxidase mutants were chronically stressed with hydrogen peroxide, the absence of glutaredoxin 1 and thioredoxin 2 did not impair growth at all, even in a minimal medium over many generations. We conclude that physiological levels of reduced oxygen species are not potent oxidants of typical protein thiols. Glutaredoxin and thioredoxin must either have an alternative purpose or else play a role under culture conditions that differ from the ones we tested.

## Introduction

**Figure 1:**
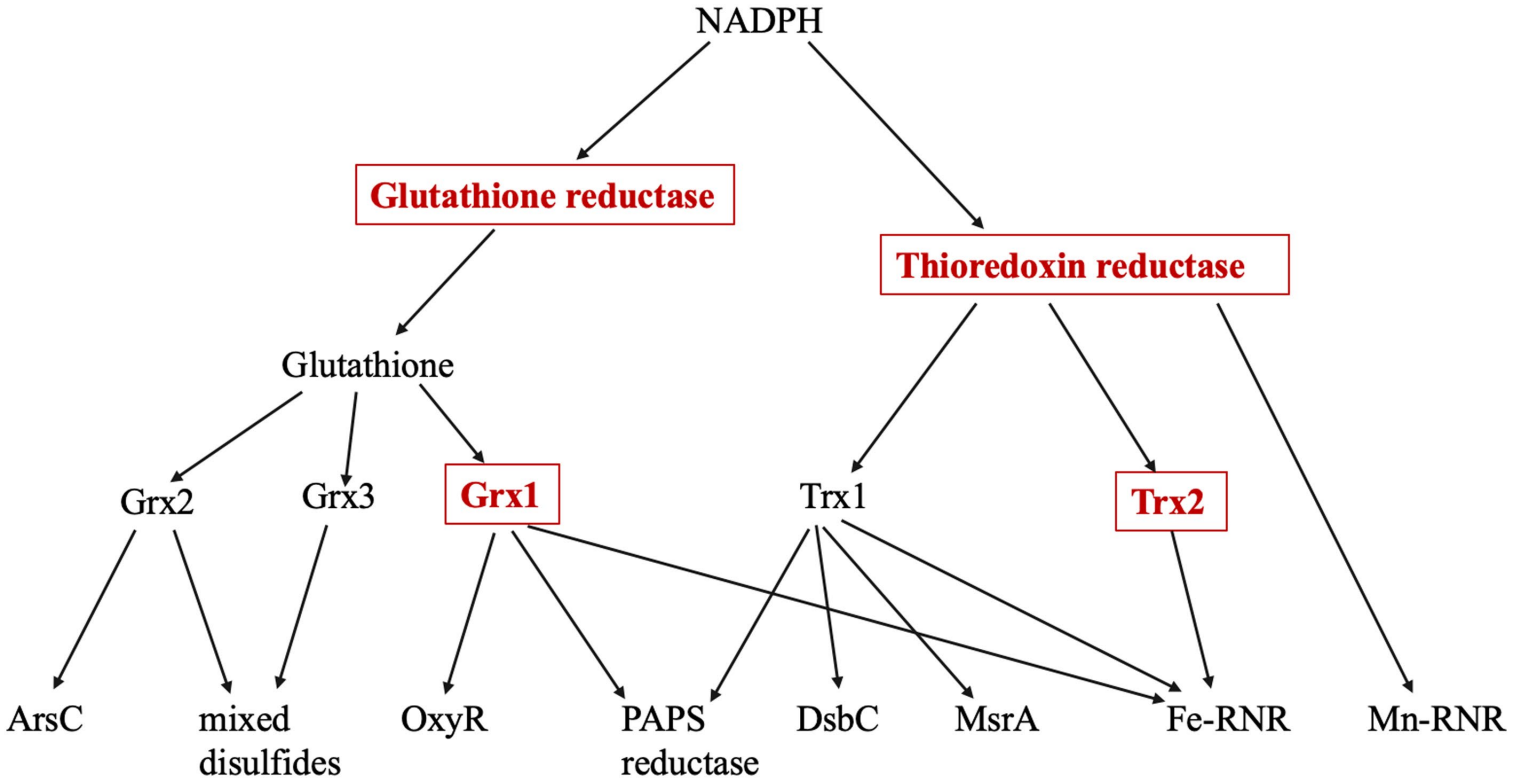
Thioredoxin and Glutaredoxin pathways. The four enzymes marked in red boxes are induced by the OxyR regulon. Glutathione reductase (*gor*), thioredoxin reductase (*trxB*), Grx1 (*grxA*), Grx2 (*grxB*), Grx3 (*grxC*), Trx1 (*trxA*), Trx2 (*trxC*), arsenate reductase (*arsC*), methionine sulfoxide reductase (*msrA*), PAPS reductase (*cysH*), Fe-RNR (*nrdAB*), Mn-RNR (*nrdHIEF*), periplasmic protein disulfide isomerase (*dsbC*). PAPS reductase is involved in the sulfate assimilation pathway; RNR denotes isozymes of ribonucleotide reductase.

Cysteine is a uniquely reactive amino acid. While it is employed in some enzymes as a nucleophile or as a metal ligand, its most distinctive feature is its redox activity. Cysteine residues are systematically oxidized to disulfide bonds in proteins that are exported to the bacterial periplasm; these bonds stabilize proteins in an environment that can be hazardous. Structural disulfide bonds are absent from proteins in the cytoplasm; however, a small group of cytoplasmic enzymes use cysteine residues as electron donors to their substrates, with the concomitant generation of transient disulfide bonds. Ribonucleotide reductase and 3-phosphoadenylyl-sulfate (PAPS) reductase are familiar examples. The catalytic cycles of these enzymes are completed when the disulfide bonds are reduced by cellular glutaredoxins and thioredoxins. These redoxins are small proteins that themselves employ solvent-exposed cysteine residues to transfer electrons to client proteins; in turn, they acquire electrons from NADPH via glutathione/glutathione reductase or thioredoxin reductase. These routes of disulfide bond formation and resolution have been worked out most completely in *Escherichia coli* and are depicted in Figure 1.

It seems unlikely that *E. coli* synthesizes two thioredoxins (encoded by *trxA* and *trxC*) and three glutaredoxins (*grxA, grxB*, and *grxC*) to reduce a mere handful of disulfide-dependent enzymes, and genetic analysis has confirmed that not all of these redoxins are needed for that purpose (Ritz and Beckwith, 2001). This observation raises the question of whether they have additional, discrete roles. A common suggestion is that redoxins also defend proteins against adventitious cysteine oxidations by oxygen species (Tao, 1997; Luikenhuis et al., 1998; Jakob et al., 1999; Carmel-Harel and Storz, 2000; Ritz et al., 2000; Li et al., 2004; Leichert et al., 2008; Wolf et al., 2008; Depuydt et al., 2009; Hillion and Antelmann, 2015; Xie et al., 2019). Potential oxidants might include not only molecular oxygen itself but also superoxide and hydrogen peroxide, both of which are formed as inadvertent by-products of metabolism.

A supporting piece of evidence is that *E. coli* induces glutaredoxin 1 and thioredoxin 2 when the bacterium is exposed to environmental hydrogen peroxide (H_2_O_2_) (Tao, 1997; Ritz et al., 2000). Hydrogen peroxide is produced in bacterial habitats by chemical events at oxic/anoxic interfaces, by photochemistry, by lactic acid bacteria, and as a major component of the cell-based immune response (Glass et al., 1986; Mehdy, 1994; Bedard et al., 2007). Because it is small and uncharged, it readily passes through membranes into cells (Lynch and Fridovich, 1978; Seaver and Imlay, 2004). Virtually all organisms possess scavenging enzymes—a mixture of catalases and peroxidases—to keep intracellular H_2_O_2_ scant, but concentrations can still become hazardous if the rate of influx is high enough. Studies of *E. coli* mutants that lack scavenging enzymes have identified two classes of metalloenzymes that H_2_O_2_ damages. It can inactivate dehydratases by oxidizing their catalytic [4Fe-4S] clusters, which then fall apart. Damage of this type affects aconitase and isopropylmalate isomerase, for example, and thereby impairs the TCA cycle and the synthesis of branched-chain amino acids (Jang and Imlay, 2007). Peroxide also reacts with mononuclear enzymes that use a single Fe(II) cofactor; the pentose-phosphate pathway and the synthesis of aromatic amino acids both rely on such enzymes, and these pathways fail during stress (Anjem and Imlay, 2012). Cells respond to these injuries by re-building the clusters and re-metallating the mononuclear sites, and the steady-state activities of affected enzymes represents the balance between the damage and repair processes. Notably, both cluster reconstruction and remetallation involve steps that require thiols (Jang and Imlay, 2007; Anjem and Imlay, 2012).

A more obvious demand for redoxins would arise if oxidants were to directly oxidize accessible cysteine residues on protein surfaces. This rationale was suggested in the studies that found that redoxins are induced in stressed cells (Tao, 1997; Luikenhuis et al., 1998; Ritz et al., 2000; Depuydt et al., 2009). *In vitro* studies have confirmed that molecular oxygen, superoxide, and H_2_O_2_ all have the capacity to oxidize thiols, and among these H_2_O_2_ stands out (Winterbourn and Metodiewa, 1999; Winterbourn, 2016). The primary scavenging system in *E. coli*, in fact, is an NADH peroxidase (AhpCF) that uses thiol-based chemistry to reduce H_2_O_2_ to water (Seaver and Imlay, 2001; Parsonage et al., 2015). And the cytoplasmic transcription factor that senses elevated H_2_O_2_—OxyR—does so when H_2_O_2_ oxidizes its sensory cysteine (Zheng et al., 1998; Choi et al., 2001). These proteins are highly evolved to react with H_2_O_2_, but they provide a chemical argument that unwanted thiol oxidations are also plausible.

Proteomic studies have repeatedly shown that the cysteine residues of cytoplasmic proteins are oxidized inside cells that are exposed to exogenous H_2_O_2_, and those data have been cited to support the notion that protein thiols are modified during oxidative stress (Leichert and Jakob, 2004; Hochgrafe et al., 2005; Leichert et al., 2008; Wolf et al., 2008; Deng et al., 2013). However, these experiments imposed H_2_O_2_ concentrations in the millimolar range—far in excess of the sub-micromolar concentrations that pertain to model bacteria (Imlay, 2013). It would be more relevant to test protein oxidation in concentrations that are likely to obtain inside natural environments.

In this study we measured the abilities of different oxygen species to oxidize either model thiols or protein cysteine residues *in vitro*. We also used alkaline phosphatase, a disulfide-dependent enzyme that had been engineered not to be secreted, to detect thiol oxidation events inside the cytoplasm. Forcing conditions were applied using strains with exceptionally high levels of internal superoxide and H_2_O_2_. The combination of these approaches permits a higher sensitivity to disulfide-bond formation than traditional proteomics. The data indicate that chemical cysteine oxidations were rare events. This result does not explain why cells induce glutaredoxin and thioredoxin systems, and it suggests either that these redoxins have undiscovered roles or that their action is important under growth conditions other than those we employed.

## Results

### Molecular oxygen is a very slow oxidant of protein thiols

The abundance of disulfide-reducing systems inside cells has generally been rationalized by the notion that either molecular oxygen or species derived from it readily oxidize the cysteine residues of proteins. The immediate product of cysteine oxidation would be a sulfenic acid; if another cysteine residue is nearby, they can condense, forming a disulfide bond. This process can be observed in cell extracts and in preparations of purified protein. Biochemists often suppress thiol oxidation by the inclusion of chelators such as EDTA, indicating that this process is metal-catalyzed and they reverse it by inclusion of reductants such as dithiothreitol (DTT) (Cleland, 1964).

Those observations of thiol oxidation are typically made on a time scale of hours to days. For thiol oxidations to be consequential inside cells—for example, to inactivate an enzyme population to the extent that a pathway bottleneck arises—the rate of thiol oxidation must be fast, with a majority of enzyme molecules being oxidized within a cell generation. We selected alkaline phosphatase (AP) as a model protein to quantify the oxidation rate. This enzyme is normally exported to the periplasm, and its activity requires that two disulfide bonds be generated, customarily by the Dsb system (Derman et al., 1993). In its reduced form the relevant cysteine residues are likely to display the oxidizability of typical protein thiols. We denatured, reduced, and renatured purified AP, generating an inactive, reduced enzyme that could regain activity through thiol oxidation. The assay detects a signal of enzyme activation that rises from a low background, rather than the loss of an enzyme activity; therefore, signal/background provides good sensitivity. Prior experiments have shown that even a modest degree of oxidation is perceptible by a rise in its activity (Eben and Imlay, 2023).

**Figure 2:**
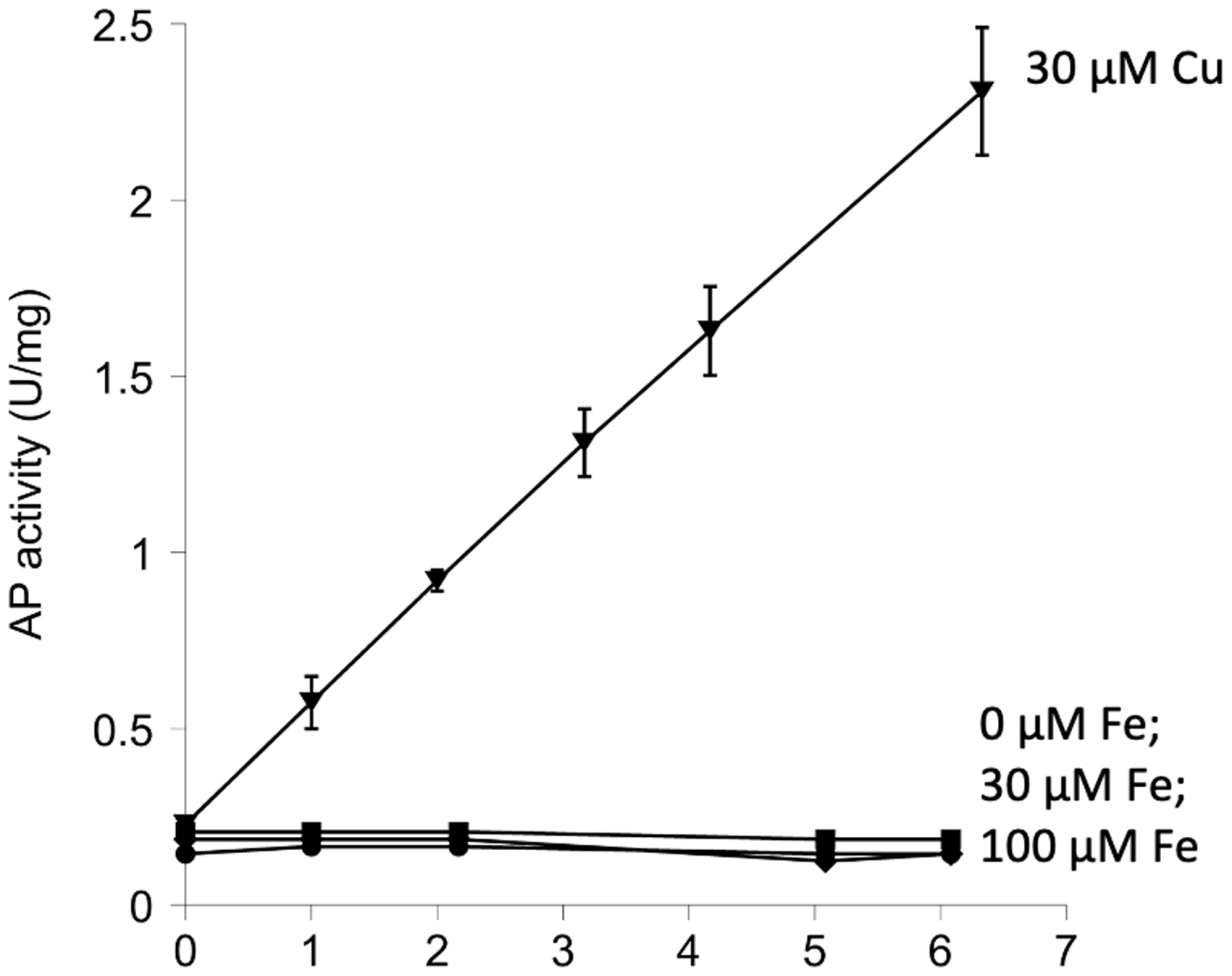
Iron does not catalyze the activation of reduced alkaline phosphatase (AP) *in vitro*. The activation of AP can be achieved by oxidation of its cysteine residues. Reduced AP was incubated at 37°C with iron (30 and 100 μM) in oxic buffers. Copper (30 μM) was provided as a positive control. In this figure and all others that follow, experiments were performed in triplicate, and error bars represent the standard error of the mean. Small error bars may be obscured by symbols.

When reduced AP was simply incubated in air-saturated buffer for as long as 6 h, no significant activation could be seen (Figure 2). Previous work showed that AP can be activated under these conditions if the system includes copper, a redox-active transition metal with high affinity for sulfur atoms (Eben and Imlay, 2023). Copper acts as a bridge that transfers electrons from cysteine side chains to oxygen. That observation was reproduced in these experiments (Figure 2). Copper, however, is excluded from the cytoplasms of bacteria (Andrei et al., 2020) and cannot drive thiol oxidation *in vivo*, so we tested whether iron might catalyze the same reaction. Loose iron is relatively abundant (20–60 micromolar) inside bacterial cytoplasms (Keyer and Imlay, 1996; Liu et al., 2011), where it is needed to metallate numerous enzymes, and it has the capacity to oxidize thiols. However, when iron was added to AP incubations, we did not observe any boost in AP activity (Figure 2).

**Figure 3:**
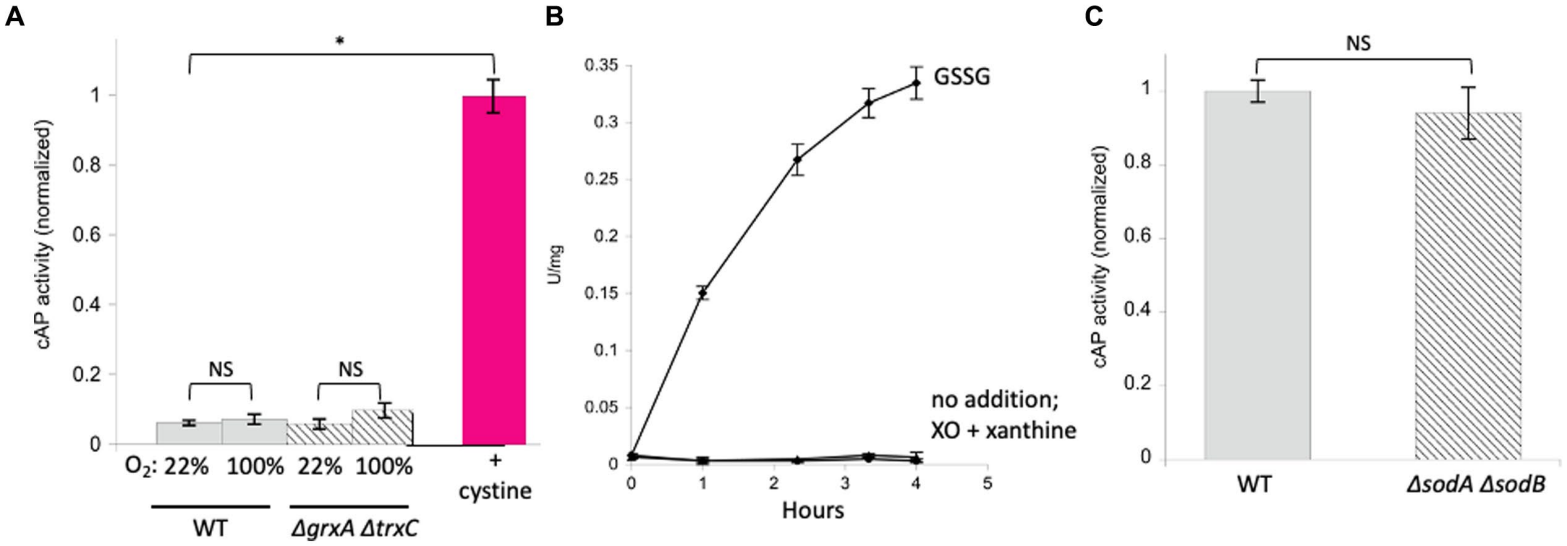
High oxygen concentrations were ineffective at activating alkaline phosphatase localized in the cytoplasm (cAP). Superoxide does not activate reduced AP *in vitro* or cAP *in vivo*. **(A)** Wild-type and *ΔgrxA ΔtrxC* strains were gassed with 22% or 100% oxygen for 40 min. The positive control was cystine (0.5 mM), the disulfide-bonded amino acid that is imported by *E. coli* and that can exchange disulfide bonds with cellular proteins. Data have been normalized to the cystine control. **(B)** Reduced AP was incubated at 37°C with xanthine oxidase and xanthine to produce superoxide. Oxidized glutathione (GSSG, 3 mM) was used as a positive control. **(C)** cAP activity was assessed in a WT strain and in a strain lacking superoxide dismutase (*ΔsodA ΔsodB*). Data have been normalized to WT; signals were as low as in Figure 1.

A more relevant test would be to monitor the activity of AP *in vivo*. We use a modified form of AP designed by Beckwith and colleagues (Derman and Beckwith, 1991). This enzyme derivative lacks a leader sequence, so the vast majority of the protein accumulates in the cytoplasm (cAP). A very small minority of the cAP harvested from cells is active, and we have shown that this background activity primarily derives from a small fraction that, despite the absence of the N-terminal leader sequence, is nevertheless exported and oxidized by the periplasmic disulfide-bond forming system (Dsb) (Eben and Imlay, 2023). To evaluate whether the majority of protein—in the cytoplasm—can be adventitiously oxidized by oxygen, we compared the amount of AP activity in cells gassed with 22% oxygen with the activity in cells that had been gassed with 100% oxygen. The data (Figure 3A) suggested a modest rise (~ 15%) in mean activity among replicate samples, but the sample-to-sample variation was large enough that statistical significance was not achieved (*p* = 0.57). The experiment was repeated in a *trxC grxA* double mutant, which lacks the inducible redoxins that are hypothesized to deal with chemical oxidation; in this strain, the apparent rise in cAP activity was larger, and the *p* value was smaller (0.20) but still in excess of the conventional line for statistical significance (Figure 3A). We tentatively conclude that hyperoxia may have a slight effect upon protein-thiol oxidation; however, because air-saturated medium has only 22% this level of oxygen, the impact in normoxic cells would be minimal. In the Discussion we consider whether the oxidation of cysteine residues might still be obscured by other routes of countervailing sulfenate/disulfide reduction.

### Superoxide does not oxidize protein thiols at a relevant pace

In a previous study we did not detect oxidation of N-acetylcysteine, a cysteine analog, when it was incubated in oxic buffer (Eben and Imlay, 2023). The apparent rate constant must therefore lie <0.3 M^−1^ s^−1^. By comparison, the rate constant for thiol oxidation by superoxide (O_2_^−^) has been estimated to be in the range of 100 M^−1^ s^−1^ (Benrahmoune et al., 2000; Winterbourn, 2016). In wild-type cells, where the steady-state O_2_-concentration is restricted by superoxide dismutase to approximately 10^−10^ M (Imlay, 2013), this rate constant is still far too low to be important, as it would project a half-time for thiol oxidation of 2 years (see calculation in Material & Methods). To test this inference, we exposed reduced AP *in vitro* to O_2_-that was steadily produced by xanthine oxidase (2 μM/min over a period of 4 h). Under these conditions the calculated steady-state level of O_2_-was 0.8 μM, but no activation was observed (Figure 3B). As this concentration is 10,000 times that of wild-type cells, we infer that even conditions that substantially elevate O_2_-production would not trigger oxidation of typical protein thiols.

Superoxide (pK_A_ = 4.8) is deprotonated at physiological pH, so its capacity as an oxidant is impaired due to the impossibility of transferring another electron onto a species that is already anionic. A more plausible oxidant is its conjugate acid HO_2_^.^, but with a pK_A_ of 4.8 it comprises only 0.25% of the superoxide at the cytoplasmic pH 7.4. The most plausible route for thiol oxidation by O_2_-is therefore when it oxidizes a thiol-bound Fe (Kawasaki et al., 2005) ion, forming a protein thiol-Fe(III)-OOH complex. For example, the standard model for bleomycin action is that an Fe(III)-OOH complex is created on the surface of DNA, from which the complex then abstracts an electron (Chow et al., 2008). We did not have a reliable way to generate such species *in vitro*, particularly because added iron can inhibit xanthine oxidase. Therefore, we tested whether O_2_-stress oxidizes cAP *in vivo*, inside cells where iron might be available.

The cAP construct was expressed in an SOD-mutant. This mutant cannot scavenge cytoplasmic O_2_^−^, and when it is aerated, the activities of superoxide-sensitive metalloenzymes immediately decline (Gardner and Fridovich, 1991; Gu and Imlay, 2013). However, we did not detect any activation of cAP (Figure 3C). The level of superoxide in this strain is extremely high, and so we conclude that O_2_-does not drive the general oxidation of protein thiols *in vivo*.

### Hydrogen peroxide can oxidize AP thiols, but only at non-physiological concentrations

Hydrogen peroxide seemed to be the best candidate to oxidize thiols, because that chemistry is used for H_2_O_2_ detection by OxyR and for scavenging by Ahp. Those two proteins are highly evolved for this function—but their rate constants for reaction with H_2_O_2_ (10^5^ M^−1^ s^−1^ for OxyR and 10^7^ M^−1^ s^−1^ for AhpC) (Zheng et al., 1998; Parsonage et al., 2015) are so high that even a lower rate for adventitious reactions might be fast enough to have physiological consequences. In line with that reasoning, workers have shown that redoxin pathways are induced when *E. coli* senses H_2_O_2_ stress. When intracellular H_2_O_2_ concentrations exceed 0.2 micromolar, the OxyR system turns on both glutathione reductase (encoded by *gor*) and glutaredoxin 1 (*grxA*), as if to enhance glutaredoxin activity, and thioredoxin 2 (*trxC*) (Christman et al., 1985; Tao, 1997; Ritz et al., 2000). These data imply that H_2_O_2_-exposed cells must amplify systems to repair oxidized cysteine residues.

The OxyR and AhpC reactions with H_2_O_2_ involve a direct attack by a deprotonated cysteine residue upon the H_2_O_2_ (Parsonage et al., 2015; Jo et al., 2017). We first tested the rate at which H_2_O_2_ oxidizes N-acetylcysteine *in vitro*. N-acetylcysteine is a cysteine analog whose amino terminus is derivatized—as is that of cysteine in polypeptides—in order to avoid effects that its positive charge might otherwise exert upon thiol behavior. A chelator was included to avoid metal-dependent reactions. A reaction was detected that was first-order in both H_2_O_2_ and the thiol (Supplementary Figure S1). The overall rate constant was low: 0.07 M^−1^ s^−1^. This experiment was conducted at pH 8, when only 3% of the thiol (pKa = 9.5) (Zhitkovich, 2019) is deprotonated; the rate constant for fully deprotonated thiolate would therefore be 2.3 M^−1^ s^−1^. This is in the range of rate constants that have been previously determined for N-acetylcysteine (Aruoma et al., 1989; Xie et al., 2019) and for free cysteine (2 M^−1^ s^−1^ at neutral pH, and 20 M^−1^ s^−1^ for the thiolate) (Winterbourn and Metodiewa, 1999) and for dithiothreitol (15 M^−1^ s^−1^ for the thiolate) (Li and Imlay, 2018). At 1 micromolar H_2_O_2_, which fully induces the OxyR system, the N-acetylcysteine rate constant implies a half-time for thiolate oxidation of 84 h—which is too long to be impactful.

**Figure 4:**
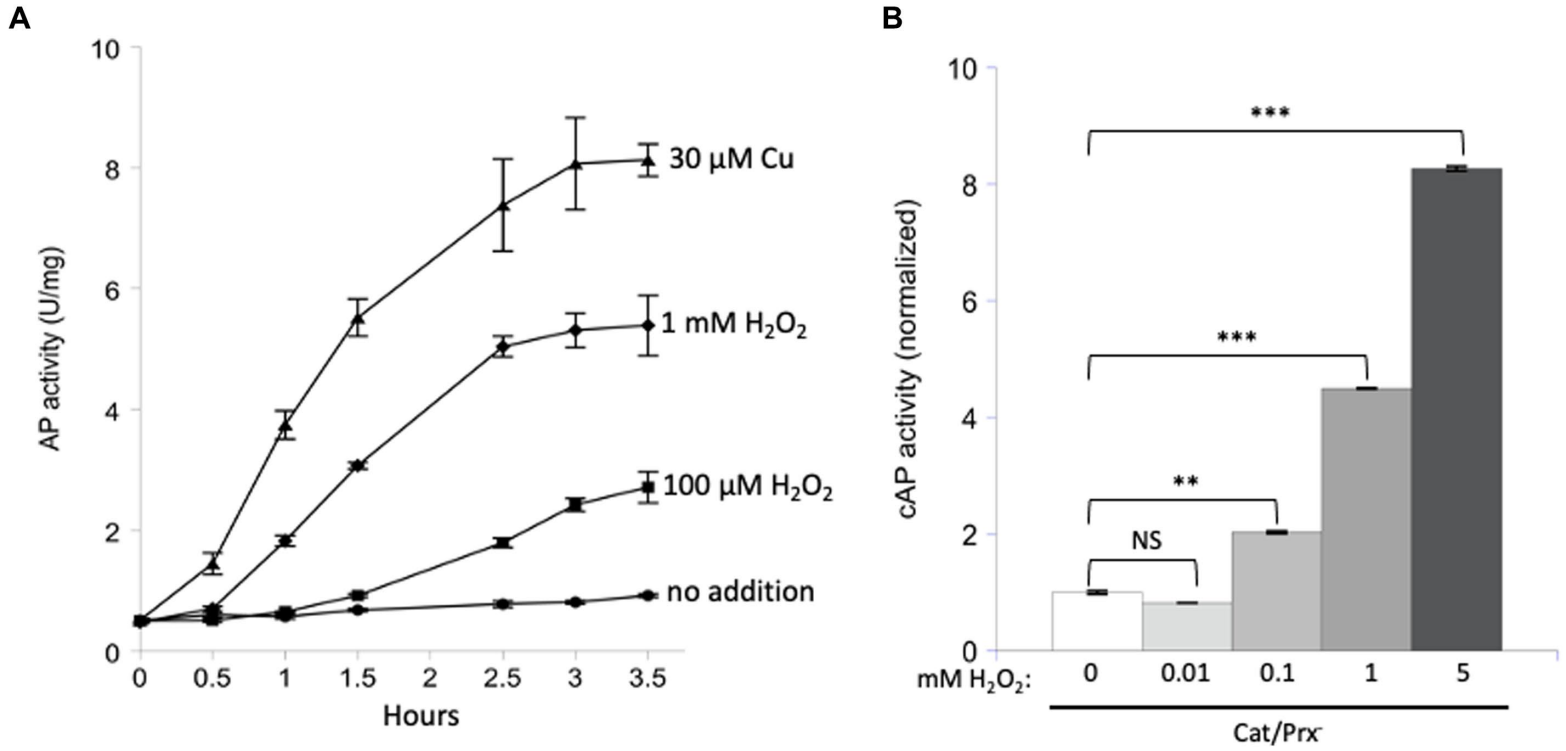
Unnaturally high concentrations of hydrogen peroxide are needed to directly activate reduced AP *in vitro* and cAP *in vivo*. **(A)** Reduced AP was incubated at 37°C with 0, 100 μM, and 1 mM H_2_O_2_ and with 30 μM copper. **(B)** cAP activity was assayed in a catalase/peroxidase-mutant exposed to a range of H_2_O_2_ concentrations (10, 100, 1, 5 mM). Dipyridyl was added to prevent metal-catalyzed oxidation, and chloramphenicol was added to stop new protein synthesis. Data have been normalized to untreated sample. For comparison: The concentration of H_2_O_2_ in WT cells has been calculated to be ~50 nM, and 1 μM cytoplasmic H_2_O_2_ fully induces the OxyR defensive response. *** indicates that *p* < 0.001; ** indicates *p* < 0.01; * indicates *p* < 0.05; NS indicates *p* > 0.05.

The experiment was repeated with AP *in vitro* (Figure 4A). Because the AP cysteine residues have evolved to be oxidized by Dsb, it seemed possible that the reaction might also be quick with H_2_O_2_. Indeed, some activation was detected, but concentrations of 100 micromolar were required.

Finally, we tested AP oxidation *in vivo*. A strain lacking scavengers of H_2_O_2_ was used, and with these cells the external and internal H_2_O_2_ concentrations are equivalent. Dipyridyl, a cell-permeable iron chelator, was initially added to prohibit iron-mediated thiol oxidation. Activation of cAP was not detected at 10 micromolar H_2_O_2_ but became apparent at 100 micromolar and quite substantial at millimolar concentrations (Figure 4B). Those data fit what had been observed *in vitro*. They also suggest that physiological levels of H_2_O_2_—which are 50 nM in unstressed cells and 200 nM during OxyR activation (Imlay, 2013)—are unlikely to directly oxidize typical cysteine residues at a consequential rate.

**Figure 5:**
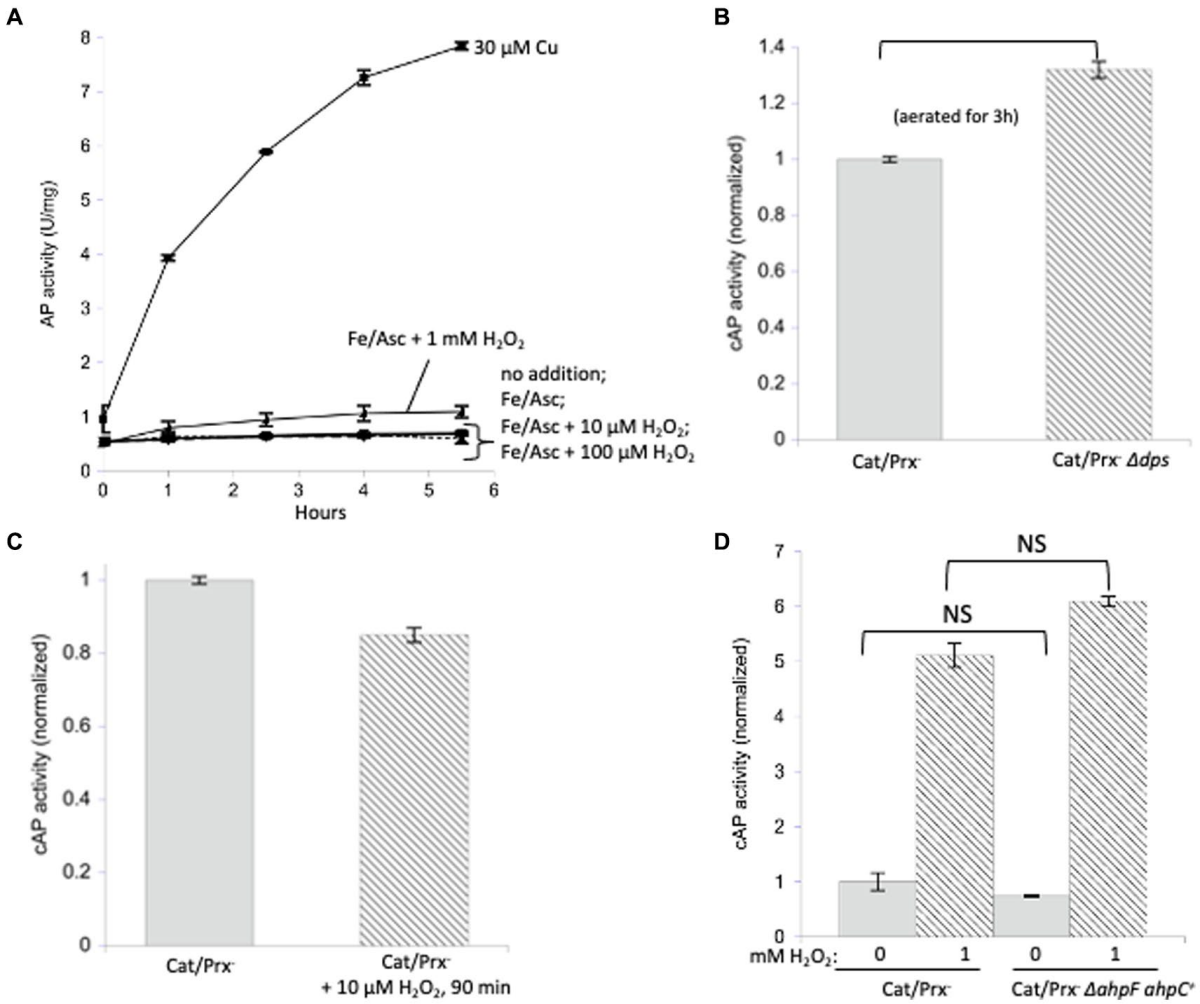
Protein disulfide bonds are not made efficiently by either iron/H_2_O_2_ Fenton chemistry or disulfide transfer from oxidized AhpC. **(A)** Reduced AP was incubated *in vitro* at 37°C with iron (50 μM) and ascorbate (10 mM) as well as increasing amounts of H_2_O_2_ (10 μM, 100 μM, 1 mM). Ascorbate ensured the constant presence of Fe (Kawasaki et al., 2005) to participate in Fenton chemistry. Control: 30 μM copper activated reduced AP. **(B)** Catalase/peroxidase-and catalase/peroxidase-Δdps strains were aerated for 3 h, and cAP activity was measured. The *dps* mutant lacks mini-ferritin and has elevated levels of intracellular free iron. Data have been normalized to the catalase/peroxidase-sample. **(C)** cAP activity did not increase in a catalase/peroxidase-mutant continuously stressed with 10 μM H_2_O_2_ over 90 min. Data have been normalized to the catalase/peroxidase-sample. **(D)** cAP activity was assessed in a catalase/peroxidase-and catalase/peroxidase-AhpC^+^ strain with and without 1 mM H_2_O_2_ for 40 min. Data have been normalized to the untreated sample.

Those results fail to explain why OxyR induces disulfide-reducing systems. One possibility might be that iron catalyzes the oxidation. We have observed this chemistry when H_2_O_2_ attacks mononuclear enzymes whose Fe (Kawasaki et al., 2005) cofactor is coordinated by a cysteine ligand: the oxidation of iron by H_2_O_2_ is unfailingly coupled to the oxidation of the cysteine residue. Because H_2_O_2_ reacts rapidly with iron, the rate constants can be as high as 10^4^ M^−1^ s^−1^ (Sobota and Imlay, 2011; Anjem and Imlay, 2012). Accordingly, we wondered whether loose iron might generally help H_2_O_2_ to oxidize protein cysteine residues. We incubated AP *in vitro* in the presence of iron and ascorbate (Figure 5A). In this system the oxidation of Fe(II) by oxygen generates O_2_-and, by dismutation, H_2_O_2_, while ascorbate reduces the oxidized Fe(III) back to Fe(II) to make the process continuous and to ensure the availability of Fe(II) to bind to enzymes. The resultant mixture of H_2_O_2_ and Fe(II) drives the Fenton reaction and has been shown to fully inactivate iron-binding enzymes (Sobota and Imlay, 2011). However, we did not observe any activation of AP.

The *in vitro* experiment may not accurately reproduce the disposition of loose iron as it occurs *in vivo*, so this model was then tested inside cells. Non-scavenging catalase/peroxidase mutants were aerated, which leads to approximately 1 micromolar H_2_O_2_ inside the cytoplasm (Mancini and Imlay, 2015). This experiment was then repeated with the additional deletion of the gene encoding Dps, an iron-sequestering miniferritin that is induced by the OxyR system. When catalase/peroxidase *dps* strains are cultured in aerobic medium, cellular levels of free iron are high, and substantial Fenton-mediated damage to DNA and to cell proteins occurs (Park et al., 2005; Anjem et al., 2009). We detected a small but statistically significant (~ 30%) increase in cAP activation (Figure 5B).

We then tested whether even higher H_2_O_2_ levels might drive cAP oxidation. The catalase/peroxidase mutant was cultured for 90 min in the presence of 10 micromolar H_2_O_2_, a dose that was continuously sustained as described in Materials and methods. The cAP activity did not rise (Figure 5C). A small decline was observed, which could reflect the slower growth rate of the stressed cells and hence cAP turnover. The fact that there was no detectable oxidation by 10 micromolar H_2_O_2_—a concentration 50-fold above what is sufficient to activate OxyR—implies that intracellular H_2_O_2_ does not cause the general oxidation of protein thiols.

A final possibility was that during H_2_O_2_ stress disulfide bonds might be disseminated from oxidized AhpC to other cellular proteins. Protein–protein disulfide transfer has been reported in two specialized circumstances: in *E. coli*, when Trx1 was forced into its oxidized, disulfide form; and in yeast, when a thiol-based peroxidase transfers a disulfide bond to Yap1 transcription factor (Stewart et al., 1998; Delaunay and AI Toledano, 2000). To create similarly forcing conditions, we knocked out *ahpF*, which encodes the disulfide reductase that reduces AhpC disulfide back to its dithiol form, so that this abundant protein would accumulate intracellularly in its oxidized form. However, upon aeration the non-scavenging AhpC^+^ AhpF-strain did not activate cAP any more than did the double mutant that lacked AhpC (Figure 5D). This remained true even when 1 mM H_2_O_2_ was added. In sum, despite multiple approaches, we were unable to find conditions under which physiological doses of H_2_O_2_ generally oxidize protein thiols.

### The inductions of grxA and trxC are strong, but these genes do not play any obvious role under the H_2_O_2_ stress conditions that we employed

**Figure 6:**
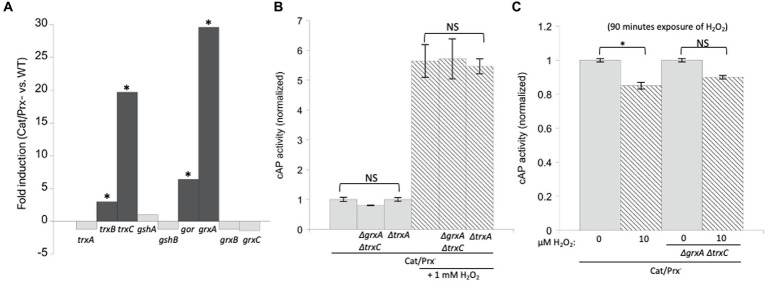
Glutaredoxin 1 (encoded by *grxA*) and thioredoxin 2 (*trxC*) are induced by H_2_O_2_ but are not needed to suppress H_2_O_2_-driven disulfide bonds. **(A)** RNA sequencing data showed that *grxA* and *trxC* are induced 30-and 20-fold, respectively, in an aerated catalase/peroxidase-strain compared to unstressed WT cells (Mancini and Imlay, 2015). The four redoxins in the dark gray bars are statistically significant. The FDR (false discovery rate adjusted *p*-values) is <100^−100^ for *gor, grxA, trxB, and trxC*. **(B)** No increase in cAP activity was observed in catalase/peroxidase-mutants lacking Grx1 and Trx2_._ Where indicated, cells were exposed to 1 mM additional H_2_O_2_ for 40 min. Data are normalized to the catalase/peroxidase-mutant. **(C)** Catalase/peroxidase-and catalase/peroxidase-ΔgrxA *ΔtrxC* strains were exposed to 10 μM H_2_O_2_ over 90 min. Data have been normalized to the respective untreated samples. *** indicates that *p* < 0.001; ** indicates *p* < 0.01; * indicates *p* < 0.05; NS indicates *p* > 0.05.

Under the conditions of our experiments, the level of H_2_O_2_ inside the catalase/peroxide mutants rises to 1–2 micromolar, and the resultant activation of OxyR induces *grxA* 20-fold and *trxC* 30-fold (Figure 6A). We considered the possibility that Grx1 and/or Trx2 reversed disulfide bonds in catalase/peroxidase mutants so rapidly that cAP activation was prevented. Therefore, we probed for cAP activation in scavenging mutants that additionally lacked both redoxins. Again, we did not see any increase in cAP activity (Figure 6B). This was true even when the constant H_2_O_2_ concentration was raised to 10 micromolar (Figure 6C). We also examined a mutant lacking Trx1, which appears to be the key disulfide reductase when disulfide bonds are introduced by agents such as diamide. It, too, had no impact upon cAP activity.

**Figure 7:**
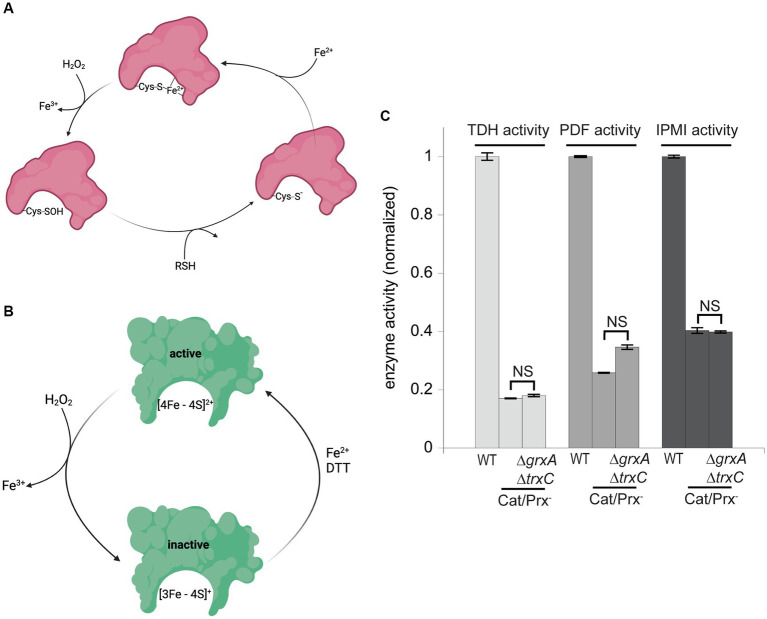
Glutaredoxin 1 and thioredoxin 2 do not aid in the repair of mononuclear iron enzymes or [4Fe-4S] cluster dehydratases. **(A)** Mononuclear iron enzymes are inactivated by H_2_O_2_. The coordinating cysteine ligand is concomitantly oxidized to a sulfenic acid; repair *in vitro* requires a thiol compound to reduce the sulfenate adduct prior to remetallation. **(B)** Dehydratase [4Fe-4S] clusters are oxidized by H_2_O_2_, and a dithiol compound is needed for cluster reactivation *in vitro*. **(C)** The mononuclear enzymes threonine dehydrogenase (TDH) and peptide deformylase (PDF), and the [4Fe-4S] dehydratase isopropylmalate isomerase (IPMI), were substantially inactivated by the ambient 1 μM H_2_O_2_ in catalase/peroxidase-mutants. Activities were not further diminished by the addition of *ΔgrxA ΔtrxC* mutations. Data have been normalized to the respective WT control. NS indicates *p* > 0.05.

These results suggested that during H_2_O_2_ stress Grx1 and Trx2 provide an action other than the reduction of typical cysteine residues. OxyR-inducing doses of H_2_O_2_ are known to damage enzymes through two mechanisms: the destruction of [4Fe-4S] clusters in dehydratases, and the oxidation of Fe (Kawasaki et al., 2005) cofactors in mononuclear enzymes (Jang and Imlay, 2007; Anjem and Imlay, 2012; Figures 7A,B). As mentioned, the oxidation of the Fe (Kawasaki et al., 2005) cofactor in mononuclear enzymes causes the co-oxidation of any cysteine ligand. This occurs in the cases of both threonine dehydrogenase (Tdh) and peptide deformylase (Pdf), each of which has a single cysteine ligand that is quantitatively oxidized when H_2_O_2_ reacts with the iron atom. When these enzymes are isolated after H_2_O_2_ stress, *in vitro* or *in vivo*, they can be reactivated only if the cysteine residue is reduced so that iron can re-bind. Both DTT and TCEP suffice *in vitro* (Anjem and Imlay, 2012); we wondered whether Grx1 or Trx2 might serve this purpose *in vivo*. Accordingly, we tracked the activities of Tdh and Pdf when catalase/peroxidase mutants were cultured in aerobic medium. Under these conditions the steady-state level of enzyme activity represents the balance between damage and repair. Tdh activity declined by ~85% and Pdf activity by ~70% in the catalase/peroxidase mutant (Figure 7C). Strikingly, the additional absence of Grx1/Trx2 had no impact, indicating that they are not needed for the reactivation process.

Cluster oxidation in dehydratases yields an inactive [3Fe-4S] cluster that lacks its substrate-binding iron atom (Figure 7B). The repair of this damage requires the provision of an electron and then an atom of ferrous iron. This process occurs with a half-time of about 5 min *in vivo*, and it can be replicated *in vitro* by incubating the damaged enzyme with Fe (Kawasaki et al., 2005) and DTT (Djaman et al., 2004). Intriguingly, the reactivation does not succeed if a monothiol is substituted for DTT, suggesting that perhaps DTT not only provides an electron but additionally helps to rearrange the cluster-binding cysteine ligands during conversion from the [3Fe-4S] to the [4Fe-4S] form. If true, it seemed plausible that the redoxins might fulfill the DTT role *in vivo*. Isopropylmalate isomerase (Jang and Imlay, 2007) was examined. Again, activity was low in catalase/peroxidase mutants (Figure 7C). The absence of Grx1 and Trx2 had no further effect. It seems clear that these two redoxins are not needed for the repair of either of the two known families of H_2_O_2_-sensitive enzymes.

**Figure 8:**
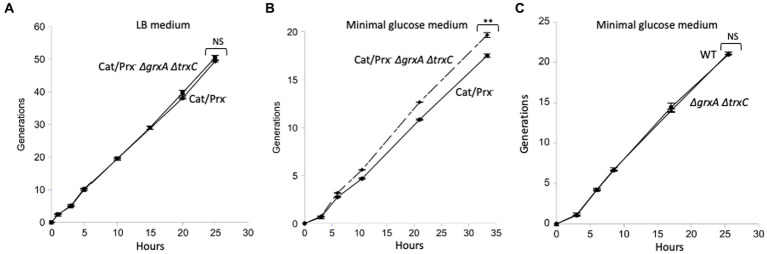
The *ΔgrxA ΔtrxC* mutations do not confer a growth disadvantage upon catalase/peroxidase-mutants. **(A)** Catalase/peroxidase-strains were cultured for 50 generations in LB medium. **(B)** The same strains were grown over 20 generations in minimal glucose medium; reproducibly, the *ΔgrxA ΔtrxC* mutants slightly outperformed the redoxin-proficient parent. **(C)** As a control, scavenging-proficient strains were cultured over 20 generations. ** indicates *p* < 0.01; * indicates *p* < 0.05; NS indicates *p* > 0.05.

In a final effort to detect any value to Grx1 and Trx2 induction, we conducted a competition experiment in which a catalase/peroxidase Grx1-Trx2^−^ mutant competed with its catalase/peroxidase Grx1^+^ Trx2^+^ sibling. Simple growth curves did not reveal apparent differences in either rate or final biomass of the two strains. A more sensitive approach is to mix the two strains and track their ratio over many generations of growth, using selective drug-resistance markers to distinguish them. We conducted the experiment both in LB medium and in a simple glucose medium; periodic dilutions kept the cell densities below 0.2 OD_600_ so that a slower strain would not have the opportunity to catch up in stationary phase. The data are shown in Figure 8.

In LB medium the two strains competed exactly evenly for 50 generations (Figure 8A). The mutant share of the population declined by <1%, indicating that over 50 generations its growth rate was >99.9% that of its GrxA^+^ TrxC^+^ parent. In the minimal glucose medium, in which cells were required to synthesize all their amino acids and cofactors, the *grxA trxC* mutant slightly but consistently outcompeted its GrxA^+^ TrxC^+^ parent (Figure 8B). This phenotype was absent from the catalase/peroxidase-proficient parent, showing that it depends upon H_2_O_2_ stress (Figure 8C). In the Discussion we suggest ideas to explain this contrarian result. In any case, it appears that Grx1 and Trx2 do not play important roles when cells are stressed with H_2_O_2_ under the standard growth conditions that we used.

## Discussion

It has frequently been proposed that oxidative stress will generate protein disulfide bonds that can disable enzymes and aggregate proteins (McDuffee et al., 1997). In this study we applied forcing conditions in order to sharply elevate the intracellular concentrations of molecular oxygen, superoxide, hydrogen peroxide, and hydroxyl radicals in a model bacterium, but our analysis found little evidence for the oxidation of cysteine residues. *In vitro* experiments appear to explain this outcome, as they indicated that these oxidants react too slowly with typical protein thiols to constitute a threat during natural levels of stress.

In mammalian cells an indirect route of protein oxidation exists. Those cells contain glutathione peroxidase (Gpx), which scavenges hydrogen peroxide. In oxidant-stressed cells Gpx turnover produces oxidized glutathione, which through exchange reactions can introduce disulfide bonds into cellular proteins. However, in bacteria the primary scavenging enzyme is AhpCF, an NADH peroxidase, and so the Gpx-dependent pathway of disulfide bond formation is absent. Instead, any formation of disulfide bonds would depend upon the direct oxidation of thiols. Our *in vivo* data, however, showed little sign that oxygen species have this effect. We will consider each species in turn.

### Reactive oxygen species do not oxidize thiols quickly

Molecular oxygen, a diradical species, is constrained to accept electrons one at a time from potential donors—and its univalent reduction potential (−0.16 V) is so low that this reaction must be slow (Imlay, 2013). The rate can be amplified by transition metals, and we and others have documented metal-driven oxidation of enzyme thiols *in vitro* and in the bacterial periplasm (Hiniker et al., 2005; Lippa and Goulian, 2012; Eben and Imlay, 2023). Copper in particular accelerates the reaction. However, efflux pumps successfully keep copper out of the bacterial cytoplasm, and iron, which is relatively abundant there, does not complex with thiols nearly as well.

From a thermodynamic view superoxide is a far stronger oxidant (Eo’ = + 0.94 V) than molecular oxygen, but because it is already an anion at physiological pH, it is unable to accept another electron. Accordingly, the rate constant for its oxidation of N-acetylcysteine was measured to be only 67 M^−1^ s^−1^; the mechanism of this reaction may not be simple electron transfer (Benrahmoune et al., 2000). Because superoxide dismutase keeps endogenous cytoplasmic O_2_-scant (10^−10^ M), this rate constant is at least three orders of magnitude too low to threaten the status of cellular thiols. In contrast, O_2_-is an efficient oxidant of metalloenzymes (10^4^ M^−1^ s^−1^), presumably because anionic O_2_-forms a stable electrostatic complex with cationic iron cofactors (Kuo et al., 1987; Flint et al., 1993). It seems likely that a momentary protonation event then triggers electron transfer, thereby generating a Fe(III)-OOH species, which ultimately decomposes to Fe(III) and H_2_O_2_. Interestingly, when this reaction was studied in enzymes whose Fe(II) atoms are liganded by a cysteine residue, the cysteine ligand itself was left untouched by the nascent Fe(III)-OOH (Gu and Imlay, 2013). Therefore, the results of this work provide no mechanism by which superoxide could directly or indirectly oxidize cysteine moieties at a relevant rate *in vivo*.

The direct oxidation of thiol compounds by H_2_O_2_ is a divalent event, initiated when a thiolate anion attacks an oxygen atom. A sulfenic acid (RSOH) is formed, and a hydroxide anion is the leaving group. Thiol-based peroxidases and OxyR have evolved several features that optimize this reaction. It is believed that they place a cationic residue near the key cysteine to ensure it is deprotonated, provide a cavity that orients H_2_O_2_ for nucleophilic attack, polarize the oxygen–oxygen bond that must be split, and provide a proton donor to stabilize the hydroxide product. These adaptations allow cysteine oxidation to occur in seconds at sub-micromolar concentrations of H_2_O_2_. We wondered whether, absent these enhancements, standard thiols might still react with H_2_O_2_ at a meaningful rate, at least on a minute time scale at micromolar H_2_O_2_. This pace would entail a second-order rate constant for the thiolate of at least 1,000 M^−1^ s^−1^. Instead, in our experiments we observed a far lower rate using N-acetylcysteine, and other studies of other thiol compounds reported rates that were similar. The cysteine residues in AP exhibited the same behavior. Enzymes whose chemistry is initiated by nucleophilic cysteine residues display rate constants that are higher—50 M^−1^ s^−1^ and 20 M^−1^ s^−1^ for *E. coli* glyceraldehyde-3-phosphate dehydrogenase and isocitrate lysase, respectively (Anjem and Imlay, 2012), and 10–20 M^−1^ s^−1^ for assorted eukaryotic tyrosine phosphatases (Denu and Tanner, 1998). But even these enzymes would exhibit an oxidation half-time in micromolar H_2_O_2_ of at least 2 h. The time would be even longer *in vivo*, where substrate competes for the active site.

In an effort to identify proteins with especially oxidizable cysteine residues, proteomic studies have typically imposed millimolar concentrations of H_2_O_2_ upon cells (Leichert and Jakob, 2004; Hochgrafe et al., 2005; Leichert et al., 2008; Wolf et al., 2008; Deng et al., 2013). Both the AP data, and consideration of fundamental rate constants, indicate that such an experimental design will cause most solvent-exposed cysteine residues to be oxidized. Indeed, such MS-based studies often identify hundreds of oxidized proteins. Consideration of the H_2_O_2_ levels in unstressed aerobic cells (50 nM) and in OxyR-activated stressed cells (1 μM) argues that millimolar H_2_O_2_ concentrations are hugely excessive and will detect oxidation events that are vanishingly rare in nature. Conversely, while a minor amount of protein disulfide bonds have been detected in unstressed cells, too, there is no evidence that these were generated by reduced oxygen species.

One mechanism of cysteine oxidation has been verified at physiological H_2_O_2_ concentrations. Rate constants are very high for Fenton reactions (10^4^ M^−1^ s^−1^) (Park et al., 2005), and the ferryl or hydroxyl radicals that they form are efficient oxidants of thiols (Davies, 2005). This chemistry was observed when low micromolar H_2_O_2_ inactivated the mononuclear enzymes peptide deformylase, threonine dehydrogenase, and cytosine deaminase (Anjem and Imlay, 2012). Each of those enzymes use cysteine, histidine, and aspartate residues to bind the Fe (Kawasaki et al., 2005) cofactor, and when H_2_O_2_ oxidizes the iron atom, the oxidation of the cysteine residue occurs concomitantly. However, the adventitious binding of loose iron to typical cysteine residues on protein surfaces would be far weaker and probably rare inside a cytoplasm that distributes its limited iron pool to many surfaces.

In our view these observations sufficiently explain why even supraphysiological levels of oxygen species were ineffective at oxidizing AP thiols in our cell experiments. We must look for alternative reasons that the cell induces Grx1 and Trx2 when it senses H_2_O_2_ stress.

### Why does OxyR induce glutaredoxin 1 and thioredoxin 2?

Of course, OxyR does not induce glutaredoxin 1 and thioredoxin 2 for no reason—so we were surprised by our inability to observe any phenotype for *grxA* and *trxC* deletions during H_2_O_2_ stress. This failure stands in complete contrast to other members of the OxyR regulon. The products of *katG*, *ahpCF*, *xthA*, *dps*, *clpSA*, *fur*, *yaaA*, *mntH*, *sufABCDE*, *hemF*, *hemH*, and *ccp* either sustain the activities of oxidant-sensitive enzymes or protect the DNA during periods of H_2_O_2_ stress; accordingly, growth and/or survival defects were apparent when any of these mutations were introduced into catalase/peroxidase mutants. Not so with *grxA* and *trxC* (Table 1).

**Table 1:** Genes induced by OxyR during H_2_O_2_ stress in *E. coli*.

Gene	Activity	Fold induction in Hpx-vs. WT	Role during H_2_O_2_ stress	H_2_O_2_ phenotype?
***trxC***	Thioredoxin 2	20.	Unknown	**No**
***trxB***	Thioredoxin reductase	3.0	Reduce Trx2	**No**
***grxA***	Glutaredoxin 1	30.	Reduce OxyR	**No**
***gor***	Glutathione reductase	6.4	Reduce Grx1	**No**
*ahpCF*	NADH peroxidase	20.^a^	Scavenge H_2_O_2_	Yes
*katG*	Catalase	57.	Scavenge H_2_O_2_	Yes
*ccp*	Cytochrome *c* peroxidase	10.^b^	Use H_2_O_2_ as a terminal electron acceptor	Yes
*dps*	Mini ferritin	9.0	Sequester iron	Yes
*fur*	Repressor for iron import	3.1	Repress iron import	Yes
*yaaA*	Unknown	15.	Minimize free iron	Yes
*clpSA*	Protease adapter protein	4.1	Activate Fe-S enzymes	Yes
*sufABCDE*	Fe-S assembly	16.	Fe-S cluster assembly	Yes
*hemF*	Coproporphyrinogen III oxidase	5.7	Heme synthesis	Yes
*hemH*	Ferrochelatase	14.	Heme synthesis	Yes
*mntH*	Manganese importer	8.6	Activate mononuclear Fe enzymes	Yes
*xthA*	Exonuclease III	5.0^c^	DNA repair	Yes
*oxyS*	Non-coding RNA	261.	Unknown	Untested
*flu*	Antigen 43	53.	Cell adhesion	Untested

Hpx-means that this strain is deficient in scavenging hydrogen peroxide (ΔkatG ΔkatE ΔahpCF). For an extended discussion of the roles of these proteins, see publication: (Sen and Imlay, 2021). References for table: ^a^Zheng et al. (2001), ^b^Khademian and Imlay (2017), ^c^Gupta and Imlay (2021), rest of table: Mancini and Imlay (2015). Bolded genes are induced by OxyR and do no exhibit a phenotyope under H_2_O_2_ stress.

This result has forced us to consider several possibilities, and these are discussed below. One is that Grx1 and Trx2 are involved in repairing metalloenzymes but that alternative routes of reduction are available in well-fed cells. Both cysteine and glutathione suffice to reduce these adducts *in vitro*, and if the levels of these thiols are high in our growing cultures, then the roles of specialized redoxins may be obscured. The other redoxins (Trx1, Grx2, and Grx3) might similarly compensate. More broadly, it is possible that rapid growth *per se* compensates for damage to an enzyme population, by rapidly replacing them through ongoing synthesis. If true, perhaps a defect may emerge when cells are carbon-limited or in stationary phase. Indeed, one study reported that thioredoxin 1 (*trxA*) is induced at low growth rates via the stringent response, as if nutritional inadequacy somehow requires a boost in redoxin activity (Lim et al., 2000).

Alternatively, these redoxins may not be generalists, like Trx1, but may have specific client proteins whose function happens to be expendable in our growth medium. Precedents exist: AhpF is dedicated to the reduction of AhpC, and NrdH acts exclusively upon the NrdEF ribonucleotide reductase. The AhpF and NrdH disulfide reductases are encoded by genes in a common operon with their target proteins; in contrast, the *grxA* and *trxC* genes are each monocistronic, providing no hint of a co-evolved partner.^1^ Nevertheless, Grx1 has been identified as the primary reductant of OxyR, and so its induction during H_2_O_2_ stress may provide a quick way to turn off the response once H_2_O_2_ levels decline (Aslund et al., 1999). Further, Potamitou et al. observed that Trx2 levels are high in some mutants defective in Grx1 function and that Grx1 levels are high in other mutants lacking Trx2 (Potamitou et al., 2002). A parsimonious explanation would be that Grx1 and Trx2 collaborate in turning off OxyR, which regulates them both. Yet it seems improbable that the cell induces two redoxin systems merely to accomplish this task.

In a recent study Winterbourn and colleagues observed that H_2_O_2_ rapidly oxidizes the active-site cysteine of mammalian glyceraldehyde-3-phosphate dehydrogenase (GAPDH), specifically when solution levels of carbon dioxide are high. The rate they observed approaches the rates at which H_2_O_2_ damages metalloenzymes, so this reaction should be biologically relevant. The effect, however, may be specific to this particular mammalian enzyme. They inferred that HCO_4_^−^, the oxidizing species, is formed by reaction between H_2_O_2_ and CO_2_ within the enzyme, enabling it to quickly oxidize the key thiol and redirect carbon flux from glycolysis to the NADPH-generating pentose-phosphate pathway (Winterbourn et al., 2023). That metabolic strategy may make sense in mammalian cells, which use NADPH as the ultimate electron donor for H_2_O_2_ scavenging, but it would be pointless for *E. coli*, which relies upon NADH. In fact, in a previous study we measured a rate constant for *E. coli* GAPDH inactivation by H_2_O_2_ of only 50 M^−1^ s^−1^ in anoxic buffer that had equilibrated with a CO_2_-rich (5%) atmosphere (Anjem and Imlay, 2012).

A very different notion is that the OxyR regulon moonlights as a defense against electrophiles other than H_2_O_2_. In this view *grxA* and *trxC* were incorporated into the regulon in order to reduce disulfide bonds created not by H_2_O_2_ but by other disulfide-generating stressors. Such compounds might include neutrophil-generated species such as hypochlorous acid (HOCl), hypothiocyanous acid (HOSCN), and nitric oxide (NO). The high efficiency with which these chemicals attack protein thiols would predispose them to activate the OxyR transcription factor. One piece of supporting evidence comes from bacteria that use PerR rather than OxyR to turn on H_2_O_2_ defenses. The PerR transcription factor is triggered not by oxidation of a sensory thiol but by the oxidation of a prosthetic iron atom, in a Fenton-type reaction—an event that seems likely to be H_2_O_2_-specific (Lee and Helmann, 2006). PerR regulons include familiar H_2_O_2_ defenses like AhpCF and catalase and the miniferritin Dps, but they do not usually include thioredoxins and glutaredoxins (Sen and Imlay, 2021). Instead, PerR-containing organisms often control redoxin synthesis using separate transcription factors that seem to be activated specifically by disulfide stress. The SigR-RsrA system of *Streptomyces coelicolor* and the Spx systems of *Bacillus subtilis* and *Staphylococcus aureus* have been most closely studied (Kang et al., 1999; Nakano et al., 2003). Workers discovered those regulons by exposing cells to diamide, a synthetic chemical (Kosower et al., 1969) designed to generate intracellular disulfide bonds; H_2_O_2_ is not an effective trigger for these regulons, and the natural inducers remain unknown. The separation of disulfide and H_2_O_2_ responses in these bacteria seems to support our conclusion that these phenomena can be unlinked.

In sum, our results suggest that we know a little bit less about disulfide stress than we thought we did. Superoxide and hydrogen peroxide are not potent producers of disulfide bonds. Thioredoxins and glutaredoxins are certainly required for routine electron delivery to a short list of enzymes, but their long-suspected role in suppressing adventitious disulfide bonds has not been confirmed. Either these proteins have a role that we have not yet imagined, or else they become important under a growth condition other than the ones we examined. Further study is warranted.

## Materials and methods

### Chemicals

Medium and buffer components were purchased from Fisher Chemical. Amino acids, casamino acids (acid-hydrolyzed), copper sulfate, ferrous ammonium sulfate, cytochrome *c*, horseradish peroxidase (HRP), Amplex Red, hydrogen peroxide solution (30% w/w), bovine catalase, ascorbic acid, ovalbumin, β-mercaptoethanol, N-acetylcysteine, bovine superoxide dismutase, reduced (GSH) and oxidized (GSSG) glutathione, *E. coli* alkaline phosphatase (catalog P5931), ascorbate, p-nitrophenylphosphate, zinc(II) diacetate, guanidinium HCl, citraconate, diethylenetriaminepentaacetic acid (DTPA), EDTA, NADH, NAD^+^, recombinant *E. coli* formate dehydrogenase (FDH), rabbit L-lactate dehydrogenase, xanthine, bovine xanthine oxidase, and antibiotics were from Sigma Aldrich. Coomassie reagent was from Thermo Scientific. Formyl-Met-Ala-Ser was from Bachem.

### Strains and plasmids

The full list of strains and plasmids can be found in Supplementary Table S1. Null mutations were made using the lambda red recombinase method to replace the open reading frame (Gebendorfer et al., 2012) with a chloramphenicol resistance cassette amplified from the pKD3 template (Datsenko and Wanner, 2000). P1 transduction was used to introduce mutations into new strains (Miller, 1972). All mutations were verified by PCR and gel analysis.

### Growth conditions

Strains were grown at 37°C in either M9 medium or minimal A medium (Miller, 1972) containing 0.2% glucose, 0.2% casamino acids, 0.5 mM histidine, 0.5 mM tryptophan, 0.5 mM thiamine, 0.01% MgSO_4_, and 0.01% CaCl_2_, unless otherwise noted. The growth medium was supplemented with 200 μg/mL ampicillin to maintain pAID135. Cultures grown aerobically were shaken vigorously. Where indicated, cultures were bubbled with 22% or 100% oxygen from an O_2_ or air gas tank with the aid of a frit.

Experiments under anoxic conditions were conducted inside an anaerobic Coy chamber. The reagents were moved into the chamber while still hot (from autoclaving) to minimize dissolved oxygen, and they were further stored overnight in the chamber to enable further degassing.

Experiments were conducted with log-phase cultures. Cells were grown overnight, subcultured to low densities (~0.0125 OD_600_), and grown for at least three generations before dilution into stress conditions for subsequent measurements. Cell density was tracked by absorbance at 600 nm.

### H_2_O_2_ measurement assay

The Amplex Red (AR)/horseradish peroxidase (HRP) assay was used to track the disappearance of H_2_O_2_ when reacting with thiols (N-acetylcysteine; NAC). The HRP reaction was set up as described in Li and Imlay (2018). The excitation and emission wavelengths used to measure fluorescence using a Shimadzu RF-150 fLuorometer were 520 and 620 nm, respectively. The buffer was 50 mM KPi, pH 8.0. All reagents were dissolved and diluted in 50 mM KPi, pH 8.0.

### Preparation of reduced alkaline phosphatase *in vitro*

To prepare *E. coli* alkaline phosphatase (AP) that was reduced and inactive, the purified enzyme (200 U, 4 mg) in 50 mM Tris, pH 8.0, was denatured with 3.6 M guanidinium HCl and reduced with 25 mM β-mercaptoethanol under aerobic conditions, as described (Eben and Imlay, 2023). The AP solution was then transferred to the anaerobic chamber and held in an incubator at 37°C overnight. Activity was checked the next day. If activity was not fully eliminated, incubation was continued at 37°C. The guanidinium HCl and β-mercaptoethanol were then removed using an Amicon Ultra-0.5 (Millipore) Centrifugal Filter Device. The denatured AP (400 μL) was loaded onto a 30 kD spin column, and the samples were centrifuged at 14,000 × g at RT for 15 min, leaving 40 μL of sample. The filtrate was discarded, and 400 μL of anoxic folding buffer was added (1 mM ZnAc_2_, 1 mM MgCl_2_, 100 mM Tris, pH 8). The column was re-centrifuged as above. For protein collection, the column was then inverted and centrifuged into a fresh tube, 1,000 × g for 2 min. The volume was adjusted to 400 μL with folding buffer. The reduced, inactive enzyme typically retained ~0.1% of the original activity. It was stored on ice in the anaerobic chamber to avoid activation in air; it was stable in this form for at least 1 month.

### Activation of purified AP

Stock solutions of 100 mM copper sulfate and 50 mM GSSG were prepared in water. H_2_O_2_ stocks were made fresh daily and also diluted in water.

Unless otherwise indicated, reduced AP (diluted 1:50) was exposed to oxidizing agents in 100 mM Tris, pH 8, at 37°C. This reaction mixture also included 10 mM MgCl_2_ and 10 mM Zn (Kawasaki et al., 2005) diacetate, as zinc is a cofactor of AP. Unless otherwise indicated, reactions were conducted in air-equilibrated buffer. Where indicated, reactions included 20 U/mL superoxide dismutase and/or 30 U/mL catalase. At time points 50 μl aliquots were removed to 1 mL 1 mM p-nitrophenylphosphate in 1 M Tris, pH 8. AP activity was measured based on its ability to hydrolyze p-nitrophenylphosphate to p-nitrophenol, a chromogenic product that absorbs at 405 nm (Bessey et al., 1946). All reactants were incubated with reduced AP at 37°C, and AP was assayed at different time points at room temperature.

To test the ability of Fe (Kawasaki et al., 2005)/oxygen to activate the enzyme, 10 mM of ferrous ammonium sulfate were prepared in water and then diluted into the reaction with reduced AP.

Activation by superoxide was tested using xanthine oxidase as the superoxide source. Xanthine oxidase was diluted 1:200 from the stock suspension (from bovine milk) shortly before use. A stock of 1 mM of xanthine was prepared in 50 mM Tris, pH 8.0, and stored at room temperature; it was diluted to 50 μM in the AP reaction. The rate of O_2_-production was measured using cytochrome *c*, and the xanthine oxidase amount was adjusted so O_2_-was generated at 2 μM/min. After 15 min, another bolus of 50 μM xanthine was added to extend the reaction. AP activity was measured at different time points.

Activation of AP with iron and ascorbate was tested in air-saturated buffer. Stock solutions of 10 mM ferrous ammonium sulfate and 100 mM ascorbate were prepared in 1 M Tris, pH 8.0. Fifty μM of ferrous ammonium sulfate and 10 mM of ascorbate were added to reduced AP, and activity was determined at intervals.

Hydrogen peroxide was also tested. H_2_O_2_ stock solution (30%) was diluted in water immediately before the experiment. Enzyme exposure to H_2_O_2_ occurred before adding zinc and magnesium. One mM ZnAc_2_ and 1 mM MgCl_2_ were added to the cuvette and incubated for 2 min before assaying. The assay buffer was 1 M Tris, pH 8.0. Time points were taken periodically.

### Activation of alkaline phosphatase in the cytoplasm

A leaderless *phoA* construct (*phoA* ∆2–22) encodes a form of alkaline phosphatase that has been used to detect disulfide bond formation in the *E. coli* cytoplasm (Derman et al., 1993). Without its signal sequence, AP accumulates in the cytoplasm, and only a tiny fraction is exported to the periplasm (Eben and Imlay, 2023). AP is only active when it acquires disulfide bonds; once those bonds are formed AP folds around them, stabilizing them against potential reductants during continued culture, cell harvesting, and extract preparation (Akiyama and Ito, 1993). AP is expressed from a pBR322-based plasmid (pAID135; Derman et al., 1993) which is ampicillin-resistant, and the *phoA* gene is under the control of a *tac* promotor. We observed that it was not necessary to induce the *tac* promoter to establish sufficient AP synthesis for our purpose, and so we did not do so, preferring steady AP production.

H_2_O_2_ and other potential oxidative stressors were added to the growth medium as described in experimental captions. To quantify AP activity inside cells, 15–20 mL of culture (at *ca.* 0.1 OD_600_) was centrifuged, resuspended in ice-cold lysis buffer (20 mM aerobic Tris pH 8.0, 10 mM EDTA), re-centrifuged, resuspended in 1 mL lysis buffer, and lysed by passage through a French press. The extract was clarified by centrifugation (17,000 × g for 20′) and then diluted 1:10 into post-lysis buffer (10 mM MgCl_2_, 10 mM Zn(II) diacetate, 1 M Tris pH 8.0). The extract was incubated for ~10 min at RT for remetallation. Then 100 μL was assayed for AP activity as above. These steps were performed using air-saturated buffers. A Bradford assay (Thermo Scientific) was used to determine the total protein concentration, with ovalbumin as the protein standard.

#### Activation of cAP in an Hpx^−^strain

Cells were grown overnight in an anaerobic chamber in minimal A medium with 0.2% glucose, 0.5 mM casamino acids, 0.5 mM tryptophan, and 200 μg/mL ampicillin. The next day cells were subcultured in the same anoxic medium and grown from OD_600_ 0.0125–0.1, subcultured again to OD_600_ 0.0125, and shaken vigorously under oxic conditions for 3 h. Cells were then harvested and cAP activity was determined.

In some experiments H_2_O_2_ was added to 10 μM. In this case the overnight cultures were diluted to OD_600_ of 0.05, grown anaerobically to an OD_600_ of 0.2, and then subcultured to OD_600_ 0.025 in air-saturated medium. The H_2_O_2_ was then added, and flasks were shaken vigorously under oxic conditions. Because H_2_O_2_ is a pseudosubstrate for the respiratory cytochrome oxidases, slow H_2_O_2_ consumption occurs in the Hpx-strain despite the absence of dedicated catalase and peroxidase enzymes. To compensate, the H_2_O_2_ concentration was measured at intervals and periodically augmented to restore the level to 10 μM. Concentrations were not allowed to fall below 8 H_2_O_2_. After 90 min the cells were harvested and cAP activity was determined.

#### Activation of cAP in a SOD^−^strain

Cells were grown overnight in an anaerobic chamber in minimal A medium with 0.2% glucose, 0.5 mM casamino acids, 0.5 mM tryptophan, and 200 μg/mL ampicillin. The overnight culture was diluted in the anaerobic chamber and cultured from OD_600_ 0.0125 to 0.1; it was then subcultured to OD_600_ 0.05 in air-saturated medium and shaken vigorously under oxic conditions for 40 min. The cellular cAP activity was then determined.

### Assay of threonine dehydrogenase

Threonine dehydrogenase is a mononuclear Fe (Kawasaki et al., 2005) enzyme that is inactivated when cellular levels of H_2_O_2_ rise. Cells were grown overnight in an anaerobic chamber in minimal A medium with 1% casamino acids (acid-hydrolyzed), 0.5 mM tryptophan, and then precultured from OD_600_ 0.0125 to 0.1. Cells were then inoculated into the same air-saturated medium and grown from OD_600_ 0.006 to 0.1 in the same aerobic medium. Fifty mL of culture was harvested. Cells were centrifuged aerobically at 6,000 rpm for 10 min at 4°C. Pellets were then moved into an anaerobic chamber, washed with ice-cold anoxic 50 mM Tris pH 8.4, and centrifuged. Pellets were then resuspended in 1 mL ice-cold lysis buffer (50 mM Tris pH 8.4, 30 mM threonine, 0.1 mM DTPA) and lysed by sonication, still inside the anaerobic chamber. Threonine was included in the lysis buffer to stabilize the iron atom in the active site. The lysate was then assayed at 340 nm for 10 min in 500 μL cuvettes, which were capped to allow anoxia to continue when cuvettes were moved outside the chamber to the spectrophotometer. The assay buffer was the same as the lysis buffer. The NAD^+^ was prepared in anoxic buffer, and 1 mM NAD^+^ was used in the assay.

### Assay of peptide deformylase

Peptide deformylase is an essential Fe (Kawasaki et al., 2005) enzyme that removes the formyl moiety from the N-terminal formylmethionine of nascent proteins. Cells were grown overnight in an anaerobic chamber in minimal A medium with 0.2% glucose, 0.2% casamino acids, and 0.5 mM tryptophan. The overnight cultures were diluted in the same anoxic medium to OD_600_ 0.0125 and cultured to OD_600_ 0.1. Cells were then diluted into the same air-saturated medium to OD_600_ 0.0125 and grown to 0.2. Fifty milliliter of culture was harvested. Cells were moved into an anaerobic chamber, centrifuged for 5 min at 6,000 rpm at 4°C, and pellets were resuspended in ice-cold anoxic 50 mM Hepes, pH 7.5, with 25 mM NaCl. Cells were resuspended in 1 mL of the same buffer and sonicated. Cell lysates were centrifuged for 2 min at 14,000 rpm at 4°C to remove debris. Lysates were then assayed at 340 nm for 10 min in 500 μL cuvettes with caps at room temperature. Reagents for the assay were prepared fresh anoxically. A typical assay consisted of 5 mM NAD^+^, 1 unit of formate dehydrogenase, and 1 mM formyl-Met-Ala-Ser tripeptide as substrate. The reaction progress was tracked by coupling formate release to NADH oxidation by formate dehydrogenase. Activity was tracked by absorbance at 340 nm.

### Assay of serine deaminase

Serine deaminase is a dehydratase dependent upon its [4Fe-4S] cluster; it converts serine into pyruvate and NH_4_^+^. Cells were grown in minimal A medium containing 0.2% glucose and 0.5 mM 18 amino acids. Overnight cultures in the anaerobic chamber were diluted to anoxic medium and grown from OD_600_ 0.0125 to 0.1. They were then diluted to OD_600_ 0.0125 in the same air-saturated medium and grown to 0.1. Fifty milliliter of culture was transferred to the anaerobic chamber. Cells were centrifuged for 8.5 min at 7,000 rpm at 4°C, and the pellet was resuspended in ice-cold anoxic 0.15 M Tris, pH 8.0, and re-centrifuged. Finally, cells were resuspended in 1 mL of the same anoxic buffer and sonicated. The lysate was centrifuged for 20 min at 4°C at 14 K rpm to remove debris. Cell extracts were assayed for 10 min in 500 μL cuvettes with caps at room temperature. A typical assay consisted of 0.15 M Tris buffer, pH 8.0, 100 mM serine, 100 μM NADH, and 10 μL LDH of a 1:100 stock. The reaction progress was tracked by coupling pyruvate formation to NADH oxidation by lactate dehydrogenase; NADH oxidation was monitored at 340 nm. NADH and LDH were prepared fresh. Serine was stored in the anaerobic chamber.

### Assay of isopropylmalate isomerase

Isopropylmalate isomerase is a dehydratase whose activity requires an intact oxidant-sensitive [4Fe-4S] cluster. Activity was measured by its action upon a pseudo substrate, citraconate. Cells were grown in minimal A medium with 0.2% glucose, 0.5 aromatic amino acids, and 0.5 mM histidine. Aromatic amino acids were provided to circumvent the inactivating effect of H_2_O_2_ upon the aromatic biosynthetic pathway (Sobota et al., 2014); histidine was provided because some MG1655 strains exhibit an anaerobic histidine bradytrophy. Anaerobic overnight cultures were diluted to 0.005 OD_600_ and grown anaerobically to 0.1 OD_600_. At that time the flasks were aerated and shaken vigorously for 2 h. Cells were then centrifuged aerobically at 4°C; the pellet was then moved to the anaerobic chamber, and all subsequent steps were performed in the anaerobic chamber with anoxic solutions. The cell pellet was resuspended in ice-cold 100 mM Tris, pH 7.6, centrifuged again, and finally resuspended in 1 mL of the same buffer. Cells were lysed by sonication, and the cell debris was removed by centrifugation at 14,000 rpm for 5 min. IPMI was promptly assayed at 235 nm in an anoxic reaction at room temperature. The enzyme gradually loses activity during storage (Jang and Imlay, 2007), so assays were performed shortly after cell lysis. Citraconate (20 mM) was used as a substrate; it was always freshly prepared, and it was dissolved in anoxic 100 mM Tris, pH 7, which was also used as the reaction buffer.

### Competition experiments

Cells were grown either in LB or M9 medium containing 0.2% glucose and 0.5 mM histidine. Overnight cultures and precultures were grown in the anaerobic chamber. Precultures were grown for at least three generations from OD_600_ 0.0125 to 0.1–0.2. Cultures grown in LB were then inoculated to OD_600_ 0.0001 in air-saturated medium, and cultures grown in M9 medium were inoculated to a starting OD_600_ of 0.001. The competing strains were mixed 1:1. Overall growth was monitored by tracking OD_600_; cultures were diluted before reaching OD_600_ of 0.3, so that the competition continued for many generations under exponential growth conditions. At different time points, aliquots were moved into the anaerobic chamber, diluted, and spread onto LB plates containing 0.2% glucose and either 12 μg/mL tetracycline or 10 μg/mL chloramphenicol, in order to quantify the viable cell number of each strain. Plates were incubated overnight at 37°C in the anaerobic chamber, and colonies were counted the next day.

### Illumina RNA sequencing

Precultures were grown in the anaerobic chamber in glucose medium to OD_600_ ~ 0.1. Cells were centrifuged 5 min at 7,000 × *g*, and pellets were resuspended in fresh oxic medium to obtain an initial OD_600_ ∼ 0.005. Cultures were incubated aerobically with vigorous shaking until they reached an OD_600_ ∼ 0.1–0.15. Total RNA was isolated from cells by hot phenol extraction (Mancini and Imlay, 2015).

### Calculations

For second-order reactions, the half-life of a reactant (with the second reactant concentration remaining constant) is described by a simple exponential equation: Ln(0.5) = −k[R_2_]t_1.2_, where k is the rate constant, [R_2_] represents the concentration of the second reactant, and t_1.2_ is the half-life. E.g., if the rate constant for N-acetylcysteine oxidation by H_2_O_2_ is 2.3 M^−1^ s^−1^, and the H_2_O_2_ concentration is 1 μM, then the half-time for NAC oxidation is 3 × 10^5^ s, or 84 h.

The steady-state concentration of O_2_-can be calculated by setting the measured rate of O_2_-formation equal to the rate of spontaneous dismutation (Imlay and Fridovich, 1991). The latter = k[O_2_^−^][HO_2_], where k = 8 × 10^7^ M^−1^ s^−1^. The pKa of superoxide is 4.8, so at pH 8 the concentration of protonated superoxide [HO_2_] = 6.3 × 10^−4^ [O_2_^−^]. Therefore, if the measured rate of O_2_-production is 2 μM/min, the steady-state O_2_-concentration is 0.8 μM.

Error bars in this paper represent the SEM of triplicate measurements. Error bars in this paper represent the SEM of triplicate measurements. Tests for statistical significance were conducted by unpaired *t*-tests using GraphPad software.^2^ *** indicates that *p* < 0.001; ** indicates *p* < 0.05; * indicates p < 0.05; NS indicates *p* > 0.05.

## Data availability statement

The original contributions presented in the study are included in the article/Supplementary material, further inquiries can be directed to the corresponding author.

## Author contributions

SE: Conceptualization, Investigation, Methodology, Writing – original draft, Writing – review & editing. JI: Funding acquisition, Resources, Writing – review & editing.

## Footnotes

^1^ https://ecocyc.org

^2^ https://www.graphpad.com

## References

[ref1] Akiyama Y. Ito K. (1993). Folding and assembly of bacterial alkaline phosphatase *in vitro* and *in vivo*. J. Biol. Chem. 268, 8146–8150. doi: 10.1016/S0021-9258(18)53073-8, PMID: 8463326

[ref2] Andrei A. Öztürk Y. Khalfaoui-Hassani B. Rauch J. Marckmann D. Trasnea P.-I.. (2020). Cu homeostasis in bacteria: the ins and outs. Membranes 10:242. doi: 10.3390/membranes10090242, PMID: 32962054 PMC7558416

[ref3] Anjem A. Imlay J. A. (2012). Mononuclear iron enzymes are primary targets of hydrogen peroxide stress. J. Biol. Chem. 287, 15544–15556. doi: 10.1074/jbc.M111.330365, PMID: 22411989 PMC3346116

[ref4] Anjem A. Varghese S. Imlay J. A. (2009). Manganese import is a key element of the OxyR response to hydrogen peroxide in *Escherichia coli*. Mol. Microbiol. 72, 844–858. doi: 10.1111/j.1365-2958.2009.06699.x 19400769 PMC2776087

[ref5] Aruoma O. I. Halliwell B. Hoey B. M. Butler J. (1989). The antioxidant action of N-acetyl cysteine: its reaction with hydrogen peroxide, hydroxyl radical, superoxide, and hypochlorous acid. Free Radic. Biol. Med. 6, 593–597. doi: 10.1016/0891-5849(89)90066-X, PMID: 2546864

[ref6] Aslund F. Zheng M. Beckwith J. Storz G. (1999). Regulation of the OxyR transcription factor by hydrogen peroxide and the cellular thiol-disulfide status. Proc. Natl. Acad. Sci. U. S. A. 96, 6161–6165. doi: 10.1073/pnas.96.11.6161, PMID: 10339558 PMC26852

[ref7] Bedard K. Lardy B. Krause K.-H. (2007). NOX family NADPH oxidases: not just in mammals. Biochimie 89, 1107–1112. doi: 10.1016/j.biochi.2007.01.012 17400358

[ref8] Benrahmoune M. Thérond P. Abedinzadeh Z. (2000). The reaction of superoxide radical with N-acetylcysteine. Free Radic. Biol. Med. 29, 775–782. doi: 10.1016/S0891-5849(00)00380-4 11053779

[ref9] Bessey O. A. Lowry O. H. Brock M. J. (1946). A method for the rapid determination of alkaline phosphates with five cubic millimeters of serum. J. Biol. Chem. 164, 321–329. 20989492

[ref10] Carmel-Harel O. Storz G. (2000). Roles of the glutathione-and thioredoxin-dependent reduction systems in the *Escherichia coli* and *Saccharomyces cerevisiae* responses to oxidative stress. Annu. Rev. Microbiol. 54, 439–461. doi: 10.1146/annurev.micro.54.1.439 11018134

[ref11] Choi H. Kim S. Mukhopadhyay P. Cho S. Woo J. Storz G.. (2001). Structural basis of the redox switch in the OxyR transcription factor. Cells 105, 103–113. doi: 10.1016/S0092-8674(01)00300-2, PMID: 11301006

[ref12] Chow M. S. Liu L. V. Solomon E. I. (2008). Further insights into the mechanism of the reaction of activated bleomycin with DNA. Proc. Natl. Acad. Sci. U. S. A. 105, 13241–13245. doi: 10.1073/pnas.0806378105, PMID: 18757754 PMC2533175

[ref13] Christman M. F. Morgan R. W. Jacobson F. S. Ames B. N. (1985). Positive control of a regulon for defenses against oxidative stress and some heat-shock proteins in *Salmonella typhimurium*. Cells 41, 753–762. doi: 10.1016/S0092-8674(85)80056-8, PMID: 2988786

[ref14] Cleland W. W. (1964). Dithiothreitol, a new protective reagent for SH groups*. Biochemistry 3, 480–482. doi: 10.1021/bi00892a002, PMID: 14192894

[ref15] Datsenko K. A. Wanner B. L. (2000). One-step inactivation of chromosomal genes in *Escherichia coli* K-12 using PCR products. Proc. Natl. Acad. Sci. U. S. A. 97, 6640–6645. doi: 10.1073/pnas.120163297, PMID: 10829079 PMC18686

[ref16] Davies M. J. (2005). The oxidative environment and protein damage. Biochim. Biophys. Acta 1703, 93–109. doi: 10.1016/j.bbapap.2004.08.007 15680218

[ref17] Delaunay A. AI Toledano M. B. (2000). H_2_O_2_ sensing through oxidation of the Yap1 transcription factor. EMBO J. 19, 5157–5166. doi: 10.1093/emboj/19.19.5157 11013218 PMC302088

[ref18] Deng X. Weerapana E. Ulanovskaya O. Sun F. Liang H. Ji Q.. (2013). Proteome-wide quantification and characterization of oxidation-sensitive cysteines in pathogenic bacteria. Cell Host Microbe 13, 358–370. doi: 10.1016/j.chom.2013.02.004, PMID: 23498960 PMC3652280

[ref19] Denu J. M. Tanner K. G. (1998). Specific and reversible inactivation of protein tyrosine phosphatases by hydrogen peroxide: evidence for a sulfenic acid intermediate and implications for redox regulation. Biochemistry 37, 5633–5642. doi: 10.1021/bi973035t, PMID: 9548949

[ref20] Depuydt M. Leonard S. E. Vertommen D. Denoncin K. Morsomme P. Wahni K.. (2009). A periplasmic reducing system protects single cysteine residues from oxidation. Science 326, 1109–1111. doi: 10.1126/science.1179557 19965429

[ref21] Derman A. I. Beckwith J. (1991). *Escherichia coli* alkaline phosphatase fails to acquire disulfide bonds when retained in the cytoplasm. J. Bacteriol. 173, 7719–7722. doi: 10.1128/jb.173.23.7719-7722.1991, PMID: 1938970 PMC212545

[ref22] Derman A. I. Prinz W. A. Belin D. Beckwith J. (1993). Mutations that allow disulfide bond formation in the cytoplasm of *Escherichia coli*. Science 262, 1744–1747. doi: 10.1126/science.8259521, PMID: 8259521

[ref23] Derman A. I. Puziss J. W. Bassford P. J. Beckwith J. (1993). A signal sequence is not required for protein export in priA mutants of *Escherichia coli*. EMBO J. 12, 879–888. doi: 10.1002/j.1460-2075.1993.tb05728.x, PMID: 8458344 PMC413286

[ref24] Djaman O. Outten F. W. Imlay J. A. (2004). Repair of oxidized iron-sulfur clusters in *Escherichia coli*. J. Biol. Chem. 279, 44590–44599. doi: 10.1074/jbc.M406487200 15308657

[ref25] Eben S. S. Imlay J. A. (2023). Excess copper catalyzes protein disulfide bond formation in the bacterial periplasm but not in the cytoplasm. Mol. Microbiol. 119, 423–438. doi: 10.1111/mmi.15032, PMID: 36756756 PMC10155707

[ref26] Flint D. H. Tuminello J. F. Emptage M. H. (1993). The inactivation of Fe-S cluster containing hydro-lyases by superoxide. J. Biol. Chem. 268, 22369–22376. doi: 10.1016/S0021-9258(18)41538-4, PMID: 8226748

[ref27] Gardner P. R. Fridovich I. (1991). Superoxide sensitivity of the *Escherichia coli* aconitase. J. Biol. Chem. 266, 19328–19333. doi: 10.1016/S0021-9258(18)55001-8 1655783

[ref28] Gebendorfer K. M. Drazic A. Le Y. Gundlach J. Bepperling A. Kastenmüller A.. (2012). Identification of a hypochlorite-specific transcription factor from *Escherichia coli*. J. Biol. Chem. 287, 6892–6903. doi: 10.1074/jbc.M111.287219, PMID: 22223481 PMC3307268

[ref29] Glass G. A. DeLisle D. DeTogni P. Gabig T. Magee B. Markert M.. (1986). The respiratory burst oxidase of human neutrophils. Further studies of the purified enzyme. J. Biol. Chem. 261, 13247–13251. doi: 10.1016/S0021-9258(18)69297-X, PMID: 3759962

[ref30] Gu M. Imlay J. A. (2013). Superoxide poisons mononuclear iron enzymes by causing mismetallation. Mol. Microbiol. 89, 123–134. doi: 10.1111/mmi.12263, PMID: 23678969 PMC3731988

[ref31] Gupta A. Imlay J. A. (2021). *Escherichia coli* induces DNA repair enzymes to protect itself from low-grade hydrogen peroxide stress. Mol. Microbiol. 117, 754–769. doi: 10.1111/mmi.14870 PMC9018492 34942039

[ref32] Hillion M. Antelmann H. (2015). Thiol-based redox switches in prokaryotes. Biol. Chem. 396, 415–444. doi: 10.1515/hsz-2015-0102, PMID: 25720121 PMC4438307

[ref33] Hiniker A. Collet J.-F. Bardwell J. C. A. (2005). Copper stress causes an *in vivo* requirement for the *Escherichia coli* disulfide isomerase DsbC*. J. Biol. Chem. 280, 33785–33791. doi: 10.1074/jbc.M505742200, PMID: 16087673

[ref34] Hochgrafe F. Mostertz J. Albrecht D. Hecker M. (2005). Fluorescence thiol modification assay: oxidatively modified proteins in *Bacillus subtilis*. Mol. Microbiol. 58, 409–425. doi: 10.1111/j.1365-2958.2005.04845.x 16194229

[ref35] Imlay J. A. (2013). The molecular mechanisms and physiological consequences of oxidative stress: lessons from a model bacterium. Nat. Rev. Microbiol. 11, 443–454. doi: 10.1038/nrmicro3032, PMID: 23712352 PMC4018742

[ref36] Imlay J. A. Fridovich I. (1991). Assay of metabolic superoxide production in *Escherichia coli*. J. Biol. Chem. 266, 6957–6965. doi: 10.1016/S0021-9258(20)89596-9 1849898

[ref37] Jakob U. Muse W. Eser M. Bardwell J. C. (1999). Chaperone activity with a redox switch. Cells 96, 341–352. doi: 10.1016/S0092-8674(00)80547-4 10025400

[ref38] Jang S. Imlay J. A. (2007). Micromolar intracellular hydrogen peroxide disrupts metabolism by damaging iron-sulfur enzymes. J. Biol. Chem. 282, 929–937. doi: 10.1074/jbc.M607646200, PMID: 17102132 PMC5136138

[ref39] Jo I. Kim D. Bang Y.-J. Ahn J. Choi S. H. Ha N.-C. (2017). The hydrogen peroxide hypersensitivity of OxyR2 in *Vibrio vulnificus* depends on conformational constraints. J. Biol. Chem. 292, 7223–7232. doi: 10.1074/jbc.M116.743765, PMID: 28264933 PMC5409488

[ref40] Kang J.-G. Paget M. S. Seok Y.-J. Hahn M.-Y. Bae J.-B. Hahn J.-S.. (1999). RsrA, an anti-sigma factor regulated by redox change. EMBO J. 18, 4292–4298. doi: 10.1093/emboj/18.15.4292, PMID: 10428967 PMC1171505

[ref41] Kawasaki S. Watamura Y. Ono M. Watanabe T. Takeda K. Niimura Y. (2005). Adaptive responses to oxygen stress in obligatory anaerobes clostridium acetobutylicum and *Clostridium aminovalericum*. Appl. Environ. Microbiol. 71, 8442–8450. doi: 10.1128/AEM.71.12.8442-8450.2005, PMID: 16332833 PMC1317462

[ref42] Keyer K. Imlay J. A. (1996). Superoxide accelerates DNA damage by elevating free-iron levels. Proc. Natl. Acad. Sci. U. S. A. 93, 13635–13640. doi: 10.1073/pnas.93.24.13635 8942986 PMC19375

[ref43] Khademian M. Imlay J. A. (2017). *Escherichia coli* cytochrome *c* peroxidase is a respiratory oxidase that enables the use of hydrogen peroxide as a terminal electron acceptor. Proc. Natl. Acad. Sci. U. S. A. 114, E6922–E6931. doi: 10.1073/pnas.1701587114 28696311 PMC5565418

[ref44] Kosower N. S. Kosower E. M. Wertheim B. (1969). Diamide, a new reagent for the intracellular oxidation of glutathione to the disulfide. Biochem. Biophys. Res. Commun. 37, 593–596. doi: 10.1016/0006-291X(69)90850-X, PMID: 5353890

[ref45] Kuo C. F. Mashino T. Fridovich I. (1987). α,β-dihydroxyisovalerate dehydratase: a superoxide-sensitive enzyme. J. Biol. Chem. 262, 4724–4727. doi: 10.1016/S0021-9258(18)61255-4, PMID: 3031031

[ref46] Lee J.-W. Helmann J. D. (2006). The PerR transcription factor senses H_2_O_2_ by metal-catalysed histidine oxidation. Nature 440, 363–367. doi: 10.1038/nature04537, PMID: 16541078

[ref47] Leichert L. I. Gehrke F. Gudiseva H. V. Blackwell T. Ilbert M. Walker A. K.. (2008). Quantifying changes in the thiol redox proteome upon oxidative stress *in vivo*. Proc. Natl. Acad. Sci. U. S. A. 105, 8197–8202. doi: 10.1073/pnas.0707723105, PMID: 18287020 PMC2448814

[ref48] Leichert L. I. Jakob U. (2004). Protein thiol modifications visualized *in vivo*. PLoS Biol. 2:e333. doi: 10.1371/journal.pbio.0020333 15502869 PMC521172

[ref49] Li K. Hein S. Zou W. Klug G. (2004). The glutathione-glutaredoxin system in *Rhodobacter capsulatus*: part of a complex regulatory network controlling defense against oxidative stress. J. Bacteriol. 186, 6800–6808. doi: 10.1128/JB.186.20.6800-6808.2004, PMID: 15466032 PMC522184

[ref50] Li X. Imlay J. A. (2018). Improved measurements of scant hydrogen peroxide enable experiments that define its threshold of toxicity for *Escherichia coli*. Free Rad. Biol. Med. 120, 217–227. doi: 10.1016/j.freeradbiomed.2018.03.025, PMID: 29550333 PMC5940505

[ref51] Lim C.-J. Daws T. Gerami-Nejad M. Fuchs J. A. (2000). Growth-phase regulation of the *Escherichia coli* thioredoxin gene. Biochim. Biophys. Acta 1491, 1–6. doi: 10.1016/S0167-4781(00)00026-9, PMID: 10760563

[ref52] Lippa A. M. Goulian M. (2012). Perturbation of the oxidizing enviornment of the periplasm stimulates the PhoQ/PhoP system in *Escherichia coli*. J. Bacteriol. 194, 1457–1463. doi: 10.1128/JB.06055-11, PMID: 22267510 PMC3294871

[ref53] Liu Y. Bauer S. C. Imlay J. A. (2011). The YaaA protein of the *Escherichia coli* OxyR regulon lessens hydrogen peroxide toxicity by diminishing the amount of intracellular unincorporated iron. J. Bacteriol. 193, 2186–2196. doi: 10.1128/JB.00001-11, PMID: 21378183 PMC3133076

[ref54] Luikenhuis S. Perrone G. Dawes I. W. Grant C. M. (1998). The yeast *Saccharomyces cerevisiae* contains two glutaredoxin genes that are required for protection against reactive oxygen species. Mol. Biol. Cell. 9, 1081–1091. doi: 10.1091/mbc.9.5.1081 9571241 PMC25331

[ref55] Lynch R. Fridovich I. (1978). Permeation of the erythrocyte stroma by superoxide radical. J. Biol. Chem. 253, 4697–4699. doi: 10.1016/S0021-9258(17)30446-5, PMID: 207707

[ref56] Mancini S. Imlay J. A. (2015). The induction of two biosynthetic enzymes helps *Escherichia coli* sustain heme synthesis and activate catalase during hydrogen peroxide stress. Mol. Microbiol. 96, 744–763. doi: 10.1111/mmi.12967, PMID: 25664592 PMC4430354

[ref57] McDuffee A. T. Senisterra G. Huntley S. Lepock J. R. Sekhar K. R. Meredith M. J.. (1997). Proteins containing non-native disulfide bonds generated by oxidative stress can act as signals for the induction of the heat shock response. J. Cell. Physiol. 171, 143–151. doi: 10.1002/(SICI)1097-4652(199705)171:2<143::AID-JCP4>3.0.CO;2-O 9130461

[ref58] Mehdy M. C. (1994). Active oxygen species in plant defense against pathogens. Plant Physiol. 105, 467–472. doi: 10.1104/pp.105.2.467, PMID: 12232215 PMC159383

[ref59] Miller J. H. Experiments in molecular genetics. Cold Spring Harbor, NY: Cold Spring Harbor Laboratory (1972).

[ref60] Nakano S. Küster-Schöck E. Grossman A. D. Zuber P. (2003). Spx-dependent global transcriptional control is induced by thiol-specific oxidative stress in *Bacillus subtilis*. Proc. Natl. Acad. Sci. U. S. A. 100, 13603–13608. doi: 10.1073/pnas.2235180100, PMID: 14597697 PMC263860

[ref61] Park S. You X. Imlay J. A. (2005). Substantial DNA damage from submicromolar intracellular hydrogen peroxide detected in Hpx-mutants of *Escherichia coli*. Proc. Natl. Acad. Sci. U. S. A. 102, 9317–9322. doi: 10.1073/pnas.0502051102, PMID: 15967999 PMC1166606

[ref62] Parsonage D. Nelson K. J. Ferrer-Sueta G. Alley S. Karplus P. A. Furdui C. M.. (2015). Dissecting peroxiredoxin catalysis: separating binding, peroxidation, and resolution for a bacterial AhpC. Biochemistry 54, 1567–1575. doi: 10.1021/bi501515w, PMID: 25633283 PMC4489686

[ref63] Potamitou A. Holmgren A. Vlamis-Gardikas A. (2002). Protein levels of *Escherichia coli* thioredoxins and glutaredoxins and their relation to null mutants, growth phase, and function. J. Biol. Chem. 277, 18561–18567. doi: 10.1074/jbc.M201225200, PMID: 11893749

[ref64] Ritz D. Beckwith J. (2001). Roles of thiol-redox pathways in bacteria. Annu. Rev. Biochem. 55, 21–48. doi: 10.1146/annurev.micro.55.1.21 11544348

[ref65] Ritz D. Patel H. Doan B. Zheng M. Aslund F. Storz G.. (2000). Thioredoxin 2 is involved in the oxidative stress response in *Escherichia coli*. J. Biol. Chem. 275, 2505–2512. doi: 10.1074/jbc.275.4.2505 10644706

[ref66] Seaver L. C. Imlay J. A. (2001). Alkyl hydroperoxide reductase is the primary scavenger of endogenous hydrogen peroxide in *Escherichia coli*. J. Bacteriol. 183, 7173–7181. doi: 10.1128/JB.183.24.7173-7181.2001, PMID: 11717276 PMC95566

[ref67] Seaver L. C. Imlay J. A. (2004). Are respiratory enzymes the primary sources of intracellular hydrogen peroxide? J. Biol. Chem. 279, 48742–48750. doi: 10.1074/jbc.M408754200, PMID: 15361522

[ref68] Sen A. Imlay J. A. (2021). How microbes defend themselves from incoming hydrogen peroxide. Front. Immunol. 12:667343. doi: 10.3389/fimmu.2021.667343, PMID: 33995399 PMC8115020

[ref69] Sobota J. M. Gu M. Imlay J. A. (2014). Intracellular hydrogen peroxide and superoxide poison 3-deoxy-D-arabinoheptulosonate 7-phosphate synthase, the first committed enzyme in the aromatic biosynthetic pathway of *Escherichia coli*. J. Bacteriol. 196, 1980–1991. doi: 10.1128/JB.01573-14 24659765 PMC4010980

[ref70] Sobota J. M. Imlay J. A. (2011). Iron enzyme ribulose-5-phosphate 3-epimerase in *Escherichia coli* is rapidly damaged by hydrogen peroxide but can be protected by manganese. Proc. Natl. Acad. Sci. U. S. A. 108, 5402–5407. doi: 10.1073/pnas.1100410108, PMID: 21402925 PMC3069151

[ref71] Stewart E. J. Aslund F. Beckwith J. (1998). Disulfide bond formation in the *Escherichia coli* cytoplasm: an *in vivo* role reversal for the thioredoxins. EMBO J. 17, 5543–5550. doi: 10.1093/emboj/17.19.5543 9755155 PMC1170883

[ref72] Tao K. (1997). oxyR-dependent induction of *Escherichia coli* grx gene expression by peroxide stress. J. Bacteriol. 179, 5967–5970. doi: 10.1128/jb.179.18.5967-5970.1997, PMID: 9294462 PMC179494

[ref73] Winterbourn C. C. (2016). Revisiting the reactions of superoxide with glutathione and other thiols. Arch. Biochem. Biophys. 595, 68–71. doi: 10.1016/j.abb.2015.11.028, PMID: 27095219

[ref74] Winterbourn C. C. Metodiewa D. (1999). Reactivity of biologically important thiol compounds with superoxide and hydrogen peroxide. Free Radic. Biol. Med. 27, 322–328. doi: 10.1016/S0891-5849(99)00051-9, PMID: 10468205

[ref75] Winterbourn C. C. Peskin A. V. Kleffmann T. Radi R. Pace P. E. (2023). Carbon dioxide/bicarbonate is required for sensitive inactivation of mammalian glyceraldehyde-3-phosphate dehydrogenase by hydrogen peroxide. Proc. Natl. Acad. Sci. U. S. A. 120:e2221047120. doi: 10.1073/pnas.2221047120, PMID: 37098065 PMC10161126

[ref76] Wolf C. Hochgräfe F. Kusch H. Albrecht D. Hecker M. Engelmann S. (2008). Proteomic analysis of antioxidant strategies of *Staphylococcus aureus*: diverse responses to different oxidants. Proteomics 8, 3139–3153. doi: 10.1002/pmic.200701062, PMID: 18604844

[ref77] Xie K. Bunse C. Marcus K. Leichert L. I. (2019). Quantifying changes in the bacterial thiol redox proteome during host-pathogen interaction. Redox Biol. 21:101087. doi: 10.1016/j.redox.2018.101087 30682706 PMC6351232

[ref78] Zheng M. Åslund F. Storz G. (1998). Activation of the OxyR transcription factor by reversible disulfide bond formation. Science 279, 1718–1722. doi: 10.1126/science.279.5357.1718, PMID: 9497290

[ref79] Zheng M. Wang X. Templeton L. J. Smulski D. R. LaRossa R. A. Storz G. (2001). DNA microarray-mediated transcriptional profiling of the *Escherichia coli* response to hydrogen peroxide. J. Bacteriol. 183, 4562–4570. doi: 10.1128/JB.183.15.4562-4570.2001, PMID: 11443091 PMC95351

[ref80] Zhitkovich A. (2019). N-acetylcysteine: antioxidant, aldehyde scavenger, and more. Chem. Res. Toxicol. 15, 1318–1319. doi: 10.1021/acs.chemrestox.9b00152 PMC6757335 31046246

