# Supplementary material for: Evidence that protein thiols are not primary targets of intracellular reactive oxygen species in growing Escherichia coli
Source: Front Microbiol. 2023 Dec 13;14:1305973. doi: 10.3389/fmicb.2023.1305973 (PMC10751367; doi:10.3389/fmicb.2023.1305973)
Supplement: Supplementary file 1 [file Data_Sheet_1.pdf]

Supplementary Information for

Evidence that protein thiols are not primary targets of intracellular reactive oxygen species in growing E. coli

Stefanie S. Eben, James A. Imlay

Corresponding author: James A. Imlay
Email: jimlay@illinois.edu

This file includes:

Tables S1
Figures S1

Table S1: Strains and plasmids

Strain	Genotype	Source
MG1655	F^- wild-type	*E. coli* Genetic Stock Center
SE05	As MG1655 plus p*phoA* (pAID135)	This study
SE17	As MG1655 plus Δ*phoA1*<br>p*phoA* (pAID135)	This study
SE93	As MG1655 plus *Δ(katG17::*Tn*10)1 (ahpC-ahpF')del kan::'ahpF Δ(katE::*Tn*10)1 dps1::cat*<br>p*phoA* (pAID135)	This study
SE105	As MG1655 plus *Δ(grxA1::cat)1 Δ(trxC1::cat)1*<br>p*phoA (pAID135)*	This study
SE107	As MG1655 plus *ΔahpCF1::cat katG17::*Tn*10 Δ(katE12::*Tn*10)1*<br>p*phoA* (pAID135)	This study
SE116	As MG1655 plus *katG17::*Tn*10 Δ(katE12::*Tn*10)1 ahpF1::cat*<br>p*phoA* (pAID135)	This study
SE119	As MG1655 plus *Δ(katG17::*Tn*10)1 Δ('dsbG) Δ(ahpC-'ahpF)~Km Δ('cedA) Δ(katE12::*Tn*10)1 Δccp1*<br>p*phoA* (pAID135)	This study
SE127	As MG1655 plus *Δ(grxA1::cat)1 Δ(trxC1::cat)1 Δ(katG17::*Tn*10)1 (ahpC-ahpF')del kan::'ahpF Δ(katE::*Tn*10)1 Δccp1*	This study

SE134	As MG1655 plus *Δ(katG17::*Tn*10)1 Δ('dsbG) Δ(ahpC-'ahpF)~Km Δ('cedA) Δ(katE12::*Tn*10)1 Δccp1 gal-76::*Tn*10*	This study
SE135	As MG1655 plus *Δ(trxC1::cat)1 Δ(katG17::*Tn*10)1 (ahpC-ahpF')del kan::'ahpF Δ(katE::*Tn*10)1 ccp1::cat*	This study
SE137	As MG1655 plus *Δ(grxA1::cat)1 Δ(katG17::*Tn*10)1 (ahpC-ahpF')del kan::'ahpF Δ(katE::*Tn*10)1 ccp1::cat*	This study
SE139	As MG1655 plus *ΔsodA1 ΔsodB1* p*phoA* (pAID135)	This study
JI367	*katE12::*Tn*10 Δ(katG17::Tn10)1*	Seaver *et al.*, 2001(1)

1. L. C. Seaver, J. A. Imlay, Alkyl hydroperoxide reductase is the primary scavenger of endogenous hydrogen peroxide in *Escherichia coli*. *J Bacteriol* **183**, 7173-7181 (2001).

Plasmid

pAID135	p*phoA*, Amp^R, tac promoter	Derman *et al.*, 1993

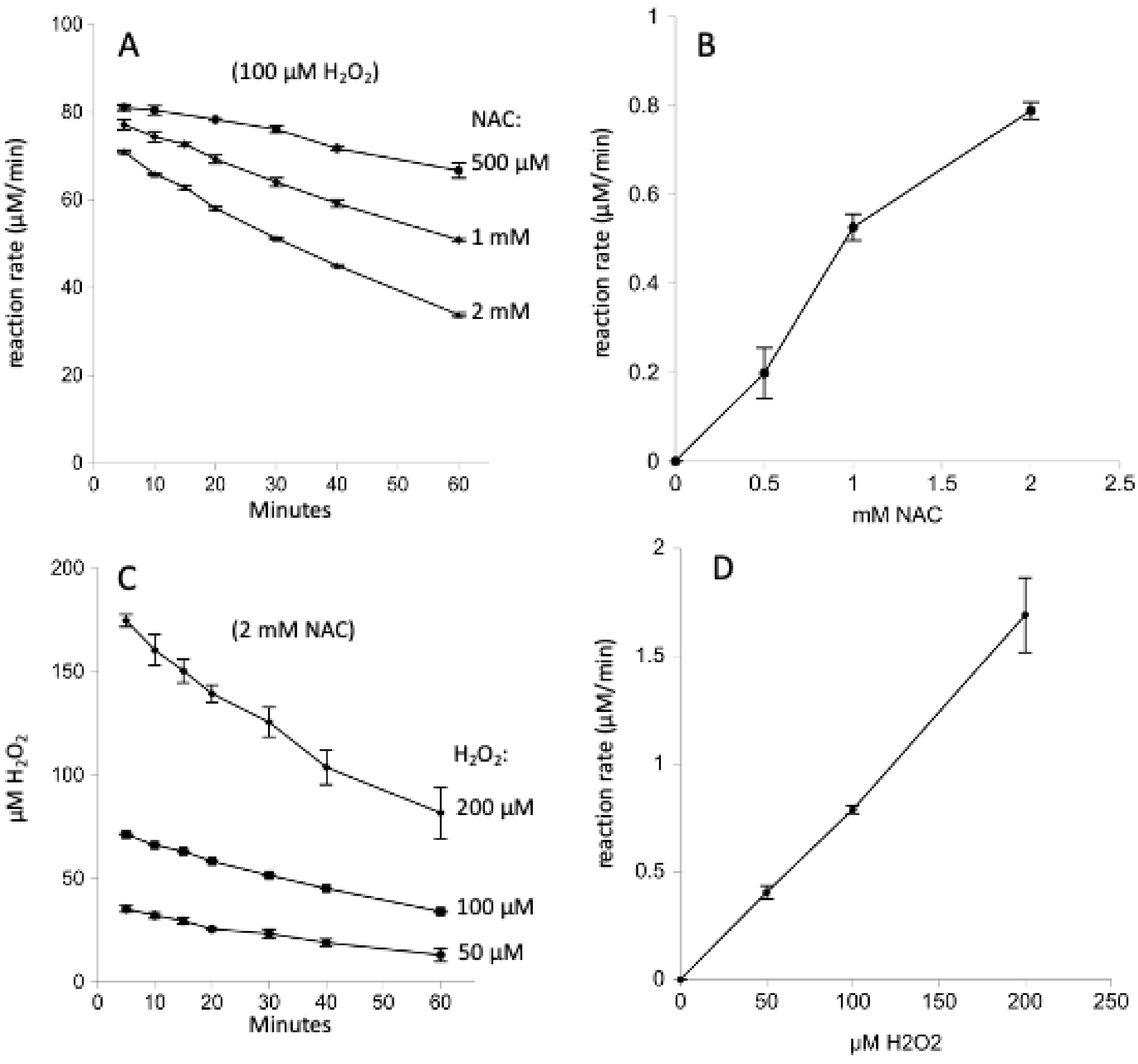

Figure S1. Hydrogen peroxide oxidizes the model thiol N-acetylcysteine (NAC) at a low rate. (A) Varying concentrations of NAC were incubated with 100 µM H_2O_2 at RT, and the reaction was tracked by the consumption of H_2O_2. (B) The rate of H_2O_2 consumption (from panel A) was proportional to NAC concentration. (C) NAC concentration (2 mM) was held constant and H_2O_2 concentration (50, 100, 200 µM) was varied. (D) The rate of thiol oxidation (from panel C) was proportional to H_2O_2 concentration. The analysis yields a rate constant for NAC oxidation of 0.07 M^{-1} s^{-1} at pH 8.
